# Reclassification of *Pterulaceae* Corner (Basidiomycota: Agaricales) introducing the ant-associated genus *Myrmecopterula* gen. nov., *Phaeopterula* Henn. and the corticioid *Radulomycetaceae* fam. nov.

**DOI:** 10.1186/s43008-019-0022-6

**Published:** 2020-01-30

**Authors:** Caio A. Leal-Dutra, Gareth W. Griffith, Maria Alice Neves, David J. McLaughlin, Esther G. McLaughlin, Lina A. Clasen, Bryn T. M. Dentinger

**Affiliations:** 1 Institute of Biological, Environmental and Rural Sciences, Aberystwyth University, Aberystwyth,, Ceredigion, SY23 3DD UK; 2 Micolab, Departamento de Botânica, Centro de Ciências Biológicas, Universidade Federal de Santa Catarina, Florianópolis, Santa Catarina Brazil; 3 Department of Plant and Microbial Biology, University of Minnesota, 1445 Gortner Avenue, St Paul, MN 55108 USA; 4 Natural History Museum of Utah & Biology Department, University of Utah, 301 Wakara Way, Salt Lake City, UT 84108 USA; 5 CAPES Foundation, Ministry of Education of Brazil, P.O. Box 250, Brasília, DF 70040-020 Brazil

**Keywords:** Molecular systematics, *Pleurotineae*, corticioid fungi, coralloid fungi, lcavarioid fungi, coral mushroom, *Aphyllophorales*, attine ants, fungus-farming ants, asexual fungi

## Abstract

*Pterulaceae* was formally proposed to group six coralloid and dimitic genera: *Actiniceps* (=*Dimorphocystis)*, *Allantula*, *Deflexula*, *Parapterulicium*, *Pterula,* and *Pterulicium*. Recent molecular studies have shown that some of the characters currently used in *Pterulaceae* do not distinguish the genera. *Actiniceps* and *Parapterulicium* have been removed, and a few other resupinate genera were added to the family. However, none of these studies intended to investigate the relationship between *Pterulaceae* genera. In this study, we generated 278 sequences from both newly collected and fungarium samples. Phylogenetic analyses supported with morphological data allowed a reclassification of *Pterulaceae* where we propose the introduction of *Myrmecopterula* gen. nov. and *Radulomycetaceae* fam. nov., the reintroduction of *Phaeopterula*, the synonymisation of *Deflexula* in *Pterulicium,* and 53 new combinations. *Pterula* is rendered polyphyletic requiring a reclassification; thus, it is split into *Pterula*, *Myrmecopterula* gen. nov., *Pterulicium* and *Phaeopterula*. *Deflexula* is recovered as paraphyletic alongside several *Pterula* species and *Pterulicium,* and is sunk into the latter genus. *Phaeopterula* is reintroduced to accommodate species with darker basidiomes. The neotropical *Myrmecopterula* gen. nov. forms a distinct clade adjacent to *Pterula*, and most members of this clade are associated with active or inactive attine ant nests. The resupinate genera *Coronicium* and *Merulicium* are recovered in a strongly supported clade close to *Pterulicium*. The other resupinate genera previously included in *Pterulaceae*, and which form basidiomes lacking cystidia and with monomitic hyphal structure (*Radulomyces*, *Radulotubus* and *Aphanobasidium*), are reclassified into Radulomycetaceae fam. nov. *Allantula* is still an enigmatic piece in this puzzle known only from the type specimen that requires molecular investigation. A key for the genera of *Pterulaceae* and *Radulomycetaceae* fam. nov. is also provided here.

## INTRODUCTION

The history of *Pterulaceae* begins with the hesitant proposal of the genus *Pterula* (hereinafter abbreviated as *Pt*.) in the early 19th century by Fries (1821, 1825, 1830). The typification of this genus was addressed by Lloyd (1919) and this was followed by discussion between Doty (1948), Donk (1949), and Rogers (1949, 1950). Ultimately Corner (1952c) provided a thorough discussion of the timeline of Fries’ decisions, which was later confirmed with further clarification by (Donk 1954; Donk 1963).

**Fig. 1:**
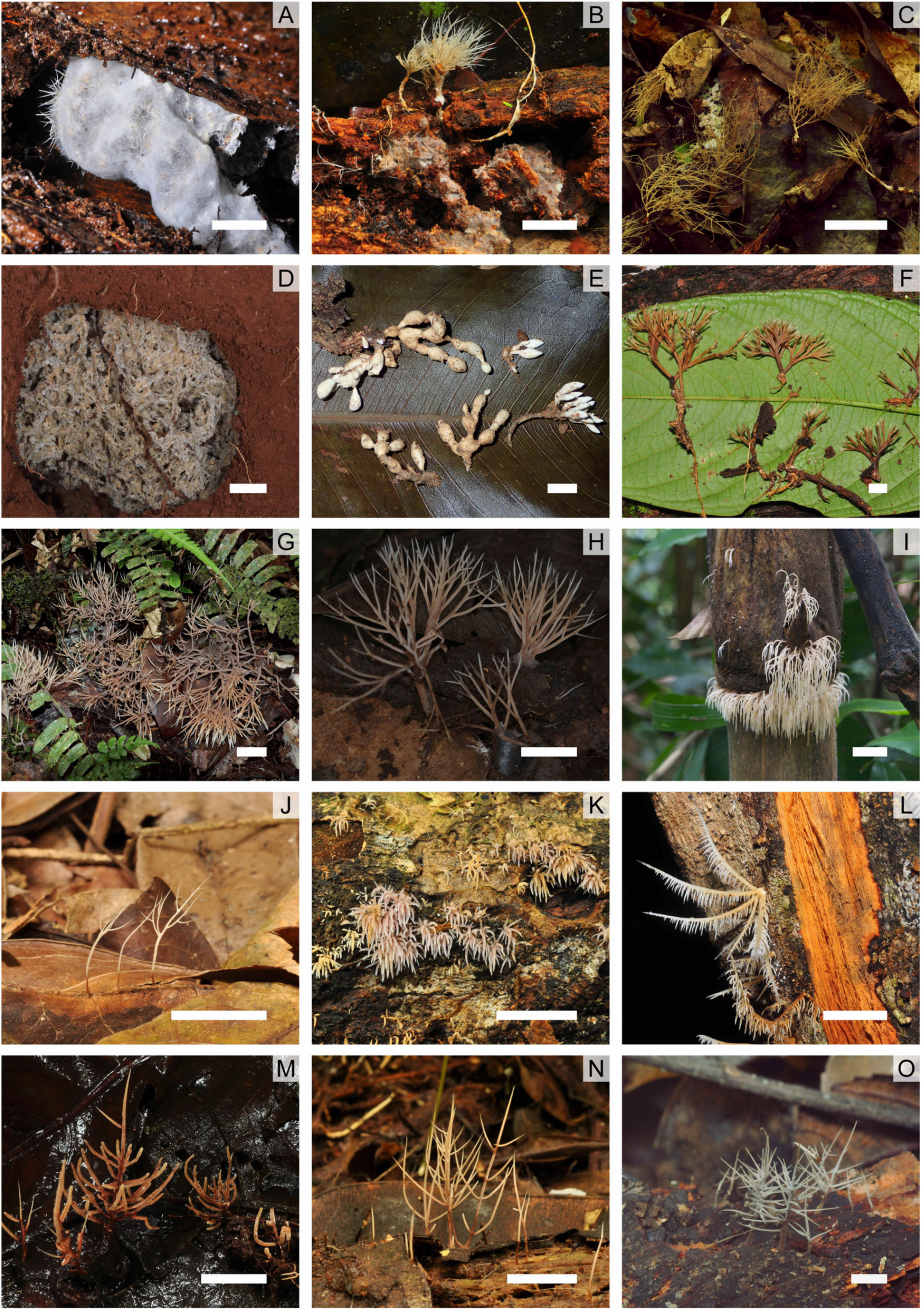
Diversity of coralloid genera of *Pterulaceae*. **a-f**: ***Myrmecopterula*** [A: *Apterostigma* sp. nest with *M. velohortorum* (RC12; CALD170307–02)* with *M.* sp*.* SAPV1 (F82; CALD170307–02)* growing on top of the garden veil; **b**: *M. sp.* (F99, HSTM-Fungos 9930); **c**: *M. sp.* (F138, FLOR 63724); **d**: *Apterostigma* sp. nest with *M. nudihortorum* (TRS111004–04)*; **e**: *M. moniliformis* (CJL585)*; **f**: *M. sp.* (F71, HSTM-Fungos 9943)]. **g-h**: ***Pterula*** [**G**: *Pt.* cf. *loretensis* (RLC273, K(M) 205,553)*; **h**: *Pt.* cf. *verticillata* (K(M) 27,119)]. **i-l**: ***Pterulicium*** [**I**: *Pm. secundirameum* (RB 575794); **j**: *Pm. aff. fluminensis* (FLOR 56379); **k**: *Pm. lilaceobrunneum* (M117, FLOR 56455). **l**: *Pm. sprucei* (F68, HSTM-Fungos 9940)]. **M-O**: ***Phaeopterula*** [**m**: *Ph. sp.* (F7, HSTM-Fungos 9944); **n**: *Ph. stipata* (M15, FLOR 56375); **o**: *Ph. juruensis* (F33, FLOR 63719)]. Close inspection of B and C reveal the basidiomes to be growing from a granular substrate resembling substrate of ants’ fungus garden. Photos **d**, **e** and **g** kindly provided by Ted Schultz, Susanne Sourell and Michael Wherley respectively. Bars = 1 cm. * Samples not deposited

The number of species in *Pterula* grew during the late 19th and early 20th centuries, with Léveille, Patouillard, Hennings, Saccardo, Lloyd, Spegazzini, and Berkeley being the most active in the naming of taxonomic novelties of *Pterula* in this period (Corner 1950, 1970). Lloyd (1919) devoted an entire chapter to discuss the taxonomy of the genus. However, the major contribution to the genus was made by E. J. H. Corner who added at least 45 new taxa (Corner 1950, 1952b, 1966, 1967, 1970). Corner (1950) created the Pteruloid series in *Clavariaceae* to group, besides *Pterula*, other genera with coralloid basidiome and dimitic hyphal system. The Pteruloid series was raised by Donk (1964) to *Pteruloideae*, a subfamily of *Clavariaceae*. *Pterulaceae* was formally proposed by Corner (1970) including the genera from the original *Pteruloideae*: *Allantula*, *Deflexula*, *Dimorphocystis* (= *Actiniceps*), *Parapterulicium*, *Pterula* and *Pterulicium* (hereinafter abbreviated as *Pm*.) (Corner 1950, 1952a, 1952b, 1970) (Fig. 1).

**Fig. 2:**
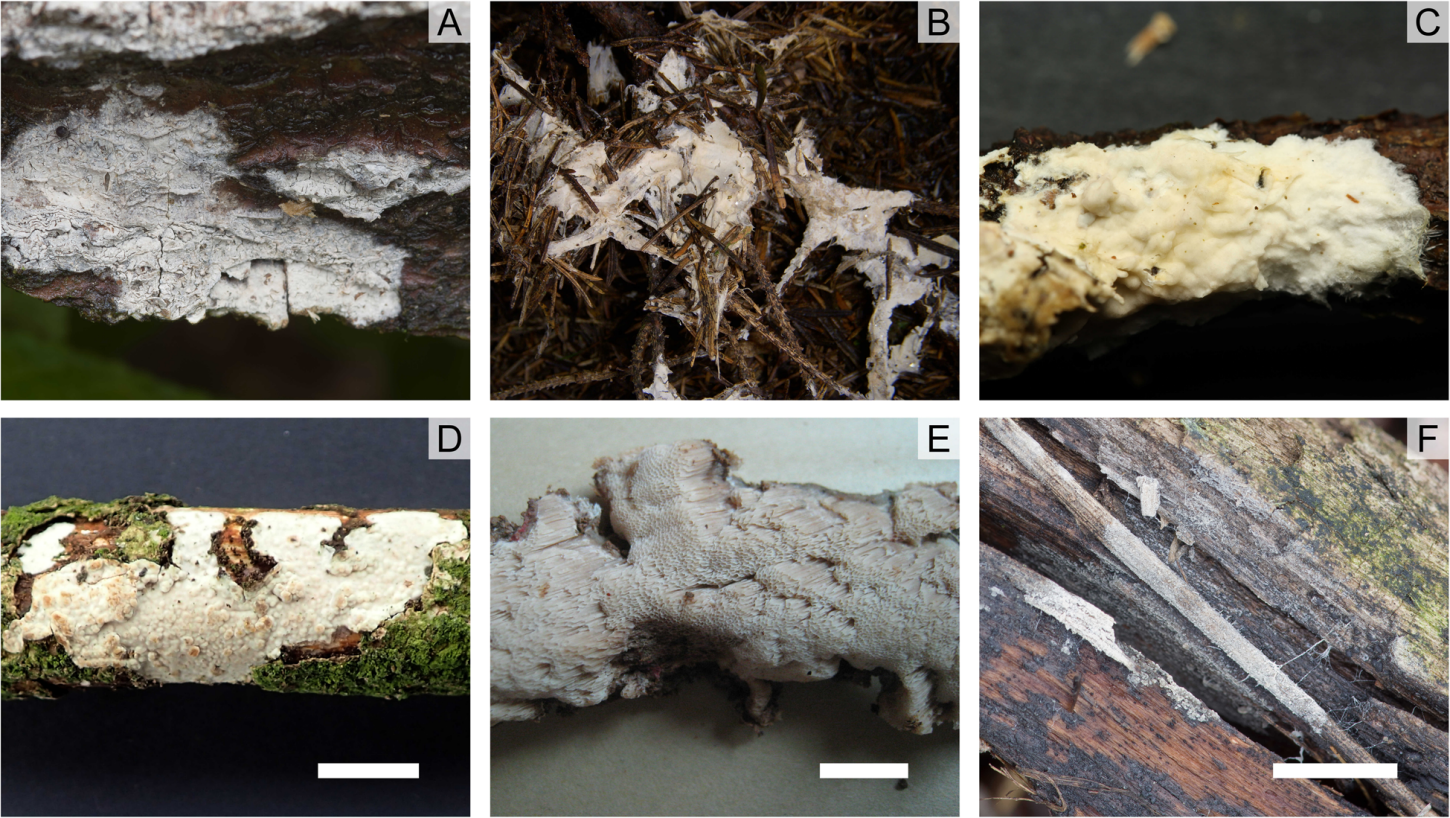
Corticioid genera of ***Pterulaceae*** (**a-c**) and ***Radulomycetaceae*** (**d-f**). **A**: Coronicium alboglaucum*. **b-c**: Merulicium fusisporum*. **d**: *Radulomyces confluens* (ABS 53). **e**: *Radulotubus resupinatus* (Dai 15,315 – BJFC). **f**: *Aphanobasidium* cf. *pseudotsugae* (ABS 54). Photos kindly provided by L. Zíbarová (**a** and **f**), S. Blaser (**b** and **c**), D.J. Harries (**d**) and C.L. Zhao (**e**). Bars = 1 cm. * Samples not deposited

Following Corner’s reclassifications, the major changes in *Pterulaceae* have resulted from molecular phylogenetic analyses. *Actiniceps* was shown within *Agaricales* to be distantly related to *Pterulaceae* and *Parapterulicium* was removed to *Russulales* (Dentinger and McLaughlin 2006; Leal-Dutra et al. 2018). Four resupinate genera were transferred to *Pterulaceae*: *Aphanobasidium*, *Coronicium*, *Merulicium*, and *Radulomyces* (Larsson 2007; Larsson et al. 2004) and, finally, the new poroid genus *Radulotubus* was proposed in the family (Zhao et al. 2016) (Fig. 2).

The ecological roles of *Pterulaceae* are not well understood, most being classified from superficial observations as saprotrophs, growing on wood or leaf litter, with wood decay potentially being the ancestral state. Whilst many species are found inhabiting soil or litter, two species are reported to associate with living plants, namely *Pterula* cf. *tenuissima*, endophytic in asymptomatic leaves of *Magnolia grandiflora*, and *Pterulicium xylogenum*, causal agent of culm rot disease of bamboo (Munkacsi et al. 2004; Villesen et al. 2004; Harsh et al. 2005) and possibly also a pathogen of sugarcane (Corner, 1952b).

*Pterulaceae* has attracted more attention recently following the discovery of two distinct symbionts of fungus-farming ants in the genus *Apterostigma* being included in several phylogenetic and ecological studies (Matheny et al. 2006; Hibbett 2007; Dentinger et al. 2009; Binder et al. 2010; Leal-Dutra 2015). Despite the absence (hitherto) of any sexual morph, phylogenetic analyses placed both species, *Pterula nudihortorum* and *Pt. velohortorum* [as G2 and G4 in Dentinger (2014)], in a strongly supported clade within *Pterulaceae* (Munkacsi et al. 2004; Villesen et al. 2004).

Whilst these earlier phylogenetic studies did not focus on resolving evolutionary relationships of the genera, they did demonstrate that the coralloid genera of *Pterulaceae* are clearly polyphyletic. Amongst the morphological characters previously used to separate the genera, but now known to be phylogenetically unreliable, is the orientation of basidiome growth that differentiates *Pterula* from *Deflexula* and the presence of a corticioid patch at the base of the basidiome in *Pterulicium* (Corner 1950, 1952a, 1970). Therefore, the reclassification of *Pterulaceae* is required to restore the monophyly of the genera.

We aimed to clarify the phylogenetic relationships of the various genera within *Pterulaceae* through collection of new samples during fieldwork campaigns in Brazil and additionally sampling of fungarium specimens. This has yielded sequence data from many specimens not included in previous phylogenetic analyses, permitting a comprehensive reappraisal of the phylogeny of *Pterulaceae*. Here we present a proposal for a new classification based on the phylogeny inferred from three nuclear loci (nrITS, nrLSU and RPB2), including representatives of all genera currently accepted in *Pterulaceae* except *Allantula*. Despite several attempts for recollecting *Allantula* in its type locality, the monotypic genus is still only known from the type specimen collected by Corner (1952a).

## METHODS

### Collections and morphological observations

Several field campaigns between 2011 and 2017 have obtained new specimens from > 15 locations in nine states across Brazil (Amazonas, Espírito Santo, Minas Gerais, Pará, Paraíba, Paraná, Rio de Janeiro, Rio Grande do Sul and Santa Catarina). The samples were dried in a low-heat food dehydrator and deposited at Aberystwyth University (ABS), Instituto Nacional de Pesquisas da Amazônia (INPA), Jardim Botânico do Rio de Janeiro (RB), Royal Botanic Gardens - Kew (K), Universidade Federal do Oeste do Pará (HSTM) and Universidade Federal de Santa Catarina (FLOR). Morphological identification and taxonomy of *Pterulaceae* are treated sensu Corner. Microscopic observations followed the methods described in Leal-Dutra (2015) and Leal-Dutra et al. (2018).

### DNA extraction, amplification, cloning and sequencing

DNA was extracted from dried basidiomes or freeze-dried cultures by first grinding with liquid nitrogen and then lysis in CTAB buffer (100 mM Tris-HCl pH 8.0, 1.4 M NaCl, 20 mM EDTA, 2% CTAB), clean-up with chloroform:isoamyl alcohol (24:1), precipitation with isopropanol (0.6 vol.) and a final wash with 70% ethanol. Partial sequences of the nrITS, nrLSU and RPB2 were amplified by PCR using the primer pairs listed on Table 1 and following the cycling conditions in the original publications. PCR products were purified using 2 U of Exonuclease I (Thermo Fisher Scientific) and 0.2 U FastAP Thermosensitive Alkaline Phosphatase (Thermo Fisher Scientific) per 1 μl of PCR product, incubated at 37 °C for 15 min, followed by heat inactivation at 85 °C for 15 min. The samples were then sent for Sanger sequencing at the IBERS Translational Genomics Facility (Aberystwyth University) or Jodrell Laboratory (Royal Botanic Gardens, Kew). The same PCR primers were used for sequencing; additional primers were used to sequence the nrLSU and RPB2 (Table 1).

**Table 1:** Primers used in this study for PCR and sequencing

Primer	Region	Application	Sequence	Reference
ITS8F	nrITS	PCR and sequencing	AGTCGTAACAAGGTTTCCGTAGGTG	(Dentinger et al. 2010)
ITS6R	nrITS	PCR and sequencing	TTCCCGCTTCACTCGCAGT	(Dentinger et al. 2010)
LR0R	nrLSU	PCR and sequencing	ACCCGCTGAACTTAAGC	(Vilgalys and Hester 1990)
LR7	nrLSU	PCR and sequencing	TACTACCACCAAGATCT	(Vilgalys and Hester 1990)
LR5	nrLSU	Sequencing	TCCTGAGGGAAACTTCG	(Vilgalys and Hester 1990)
fRPB2-5F	RPB2	PCR and sequencing	GAYGAYMGWGATCAYTTYGG	(Liu et al. 1999)
bRPB2–7.1R	RPB2	PCR and sequencing	CCCATRGCYTGYTTMCCCATDGC	(Matheny 2005)
bRPB2-6F	RPB2	Sequencing	TGGGGYATGGTNTGYCCYGC	(Matheny 2005)

Chromatograms were manually checked and sequences assembled and edited using GENEIOUS 10.0.2 (Kearse et al. 2012). Samples presenting indels were cloned using pGEM-T Easy Vector Systems (Promega) into Subcloning Efficiency DH5α Competent Cells (Invitrogen). Up to five clones from each sample were amplified and sequenced as above. For each sample clone sequences were aligned to generate one or more consensus sequences and polymorphisms were replaced by respective IUPAC code for ambiguous nucleotide; in cases where indels were found, two different sequences were saved (Leal-Dutra et al. 2018).

Moreover, 27 sequences of nrITS (4), nrLSU (10) and RPB2 (13) were mined from 13 previously assembled and unpublished genomes using NCBI BLAST+ package v2.7.1 (Camacho et al. 2009). Two sequences of each *Pterulaceae* genus were used as query and the best hit based on the combination of e-value and bit score was selected; the same hit should usually appear for all query sequences. In one case (sample KM190547), more than one optimal hit was found; the subject sequences were compared for occurrence of indels and treated as virtual clones (VC). These sequences are included in the dataset (Table 2). The sequences generated in this study have been submitted to GenBank (Table 2).

**Table 2:** Details of new sequences generated in this study used in the tree of Fig. 3. (See also Additional file 1)

Taxon (former genus in brackets)	DNA sample ID	Fungarium voucher	Country	Region	ITS	LSU	RPB2
*Coronicium alboglaucum*	K15	K(M) 170129	UK	England	MK953245	–	–
*Coronicium gemmiferum*	K13	K(M) 133847	UK	England	MK953246	–	–
*Coronicium gemmiferum*	K14	K(M) 68853	UK	England	MK953247	MK953403	–
*Merulicium fusisporum*	K16	K(M) 45181	UK	England	MK953248	–	–
*Myrmecopterula (Pterula) moniliformis*	F92 Consensus 1	INPA 280127	Brazil	Amazonas	MK953251	MK953405	MK944362
*Myrmecopterula (Pterula) moniliformis*	M39	FLOR 56397	Brazil	Paraíba	MK953253	MK953406	–
*Myrmecopterula (Pterula) moniliformis*	MCA	not deposited	–	–	MK953239	MK953392	MK944363
*Myrmecopterula (Pterula) nudihortorum*	F144 Consensus 1	not deposited	Brazil	Amazonas	MK953257	MK953393	MK944364
*Myrmecopterula (Pterula) nudihortorum*	KM190547_VC1	K(M) 190547	Panama	–	MK953240	MK953394	MK944365
*Myrmecopterula (Pterula)* sp*.*	F103	HSTM-Fungos 9931	Brazil	Pará	MK953260	MK953407	MK944325
*Myrmecopterula (Pterula) sp.*	F138	FLOR 63724	Brazil	Paraná	MK953262	MK953409	–
*Myrmecopterula (Pterula)* sp*.*	F40	FLOR 63725	Brazil	Paraná	MK953264	MK953410	MK944327
*Myrmecopterula (Pterula)* sp*.*	F82 Consensus 1	not deposited	Brazil	Amazonas	MK953269	MK953412	MK944366
*Myrmecopterula (Pterula)* sp*.*	F94	HSTM-Fungos 9928	Brazil	Pará	MK953274	MK953414	MK944329
*Myrmecopterula (Pterula)* sp*.*	F99	HSTM-Fungos 9930	Brazil	Pará	MK953276	MK953415	MK944330
*Myrmecopterula (Pterula)* sp*.*	M111	FLOR 56451	Brazil	Santa Catarina	MK953277	–	–
*Myrmecopterula (Pterula)* sp*.*	M40 Consensus 1	FLOR 56398; K(M) 205347	Brazil	Paraíba	MK953280	MK953416	MK944367
*Myrmecopterula (Pterula)* sp*.*	M69	FLOR 56418	Brazil	Rio Grande do Sul	MK953281	MK953395	MK944368
*Myrmecopterula (Pterula) velohortorum*	F114	not deposited	Brazil	Espírito Santo	MK953282	MK953396	MK944369
*Myrmecopterula (Pterula) velohortorum*	F117	not deposited	Brazil	Santa Catarina	MK953283	–	–
*Myrmecopterula (Pterula) velohortorum*	F135	not deposited	Brazil	Pará	MK953285	–	–
*Myrmecopterula (Pterula) velohortorum*	F136	not deposited	Brazil	Pará	MK953286	–	–
*Myrmecopterula (Pterula) velohortorum*	F137	not deposited	Brazil	Pará	MK953287	–	–
*Myrmecopterula (Pterula) velohortorum*	F140 Clone 1	not deposited	Brazil	Amazonas	MK953288	–	–
*Myrmecopterula (Pterula) velohortorum*	F152	not deposited	Brazil	Santa Catarina	MK953290	–	–
*Myrmecopterula (Pterula) velohortorum*	KM190546	K(M) 190546	Panama	–	MK953242	MK953397	MK944370
*Myrmecopterula (Pterula) velohortorum*	RC12 Consensus 1	not deposited	Brazil	Amazonas	MK953291	–	–
*Phaeopterula (Pterula) anomala*	KM38182	K(M) 38182	Cameroon	–	MK953295	–	–
*Phaeopterula (Pterula)* cf. *juruensis*	F45 Consensus 1	FLOR 63732	Brazil	Paraná	MK953296	MK953417	MK944331
*Phaeopterula (Pterula)* cf. *juruensis*	F79 Consensus 1	FLOR 63717	Brazil	Paraná	MK953299	MK953418	–
*Phaeopterula (Pterula)* cf. *stipata*	F66 Consensus 1	HSTM-Fungos 9938	Brazil	Pará	MK953301	–	–
*Phaeopterula (Pterula)* cf. *stipata*	F98 Consensus 1	HSTM-Fungos 9929	Brazil	Pará	MK953302	–	–
*Phaeopterula (Pterula)* cf. *taxiformis*	M4	FLOR 56367	Brazil	Santa Catarina	MK953303	MK953419	–
*Phaeopterula (Pterula) juruensis*	F41	FLOR 63728	Brazil	Paraná	MK953304	MK953420	MK944332
*Phaeopterula (Pterula) juruensis*	M21	FLOR 56381	Brazil	Minas Gerais	MK953305	–	–
*Phaeopterula (Pterula) juruensis*	M36	FLOR 56396	Brazil	Santa Catarina	MK953307	MK953422	–
*Phaeopterula (Pterula)* sp*.*	F63 Consensus 1	HSTM-Fungos 9935	Brazil	Pará	MK953316	MK953425	MK944335
*Phaeopterula (Pterula)* sp*.*	F78 Clone 1	FLOR 63716	Brazil	Paraná	MK953321	MK953428	MK944338
*Phaeopterula (Pterula)* sp*.*	KM135954	K(M) 135954	Belize	–	MK953326	–	–
*Phaeopterula (Pterula)* sp*.*	KM137475	K(M) 137475	Puerto Rico	–	MK953327	–	–
*Phaeopterula (Pterula) stipata*	M15 Consensus 1	FLOR 56375	Brazil	Minas Gerais	MK953330	MK953431	–
*Phaeopterula* sp*. (Allantula?)*	F7 Consensus 1	HSTM-Fungos 9944	Brazil	Pará	MK953331	MK953432	MK944339
*Pterula* cf *plumosa*	KM167176	K(M) 167176	Ethiopia	–	MK953333	–	–
*Pterula* cf. *loretensis*	RLC273	K(M) 205553	Ecuador	Imbabura	MK953334	MK953398	MK944371
*Pterula multifida*	KM195746	K(M) 195746	UK	England	MK953335	MK953399	MK944372
*Pterula* sp*.*	F42	FLOR 63729	Brazil	Paraná	MK953336	MK953433	–
*Pterula* sp*.*	F48	FLOR 63735	Brazil	Paraná	–	MK953434	–
*Pterula* sp*.*	M112 Consensus 1	FLOR 56452	Brazil	Santa Catarina	MK953337	MK953435	MK944340
*Pterula* sp*.*	M153	FLOR 57849	Brazil	Santa Catarina	MK953339	MK953436	MK944341
*Pterula* sp*.*	M54	FLOR 56407	Brazil	Santa Catarina	MK953341	MK953438	–
*Pterula* sp*.*	M71 Consensus 1	FLOR 56424	Brazil	Santa Catarina	MK953342	MK953439	MK944342
*Pterula* sp*.*	KM141379	K(M) 141379	Puerto Rico	–	MK953344	–	–
*Pterula* sp*.*	KM167221	K(M) 167221	Australia	Queensland	MK953345	–	–
*Pterula subulata*	KM145950	K(M) 145,950	Italy	–	MK953346	–	–
*Pterula subulata*	KM167186	K(M) 167,186	Sweden	–	MK953347	–	–
*Pterula verticillata*	KM27119	K(M) 27119	Brunei	–	MK953348	–	–
*Pterulicium (Deflexula) fasciculare*	KM167225	K(M) 167225	Australia	–	MK953349	–	–
*Pterulicium (Deflexula) fasciculare*	KM167227	K(M) 167227	Malaysia	–	MK953350	–	–
*Pterulicium (Deflexula) lilaceobrunneum*	M117	FLOR 56455	Brazil	Rio de Janeiro	MK953351	MK953440	MK944343
*Pterulicium (Deflexula) secundirameum*	BZL44	RB 575791	Brazil	Rio de Janeiro	MK953353	MK953400	MK944373
*Pterulicium (Deflexula) secundirameum*	M50	FLOR 56403	Brazil	Santa Catarina	MK953354	MK953442	MK944344
*Pterulicium (Deflexula)* sp.	KM167228	K(M) 167228	Malaysia	–	MK953357	–	–
*Pterulicium (Deflexula)* sp.	KM167233	K(M) 167233	Sierra Leone	–	MK953358	–	–
*Pterulicium (Deflexula) sprucei*	F68	HSTM-Fungos 9940	Brazil	Pará	MK953361	MK953447	MK944349
*Pterulicium (Deflexula) subsimplex*	KM160100	K(M) 160100	Ecuador	–	–	MK953449	–
*Pterulicium (Deflexula) subsimplex*	M33	FLOR 56393	Brazil	Santa Catarina	MK953363	MK953450	MK944351
*Pterulicium (Pterula) brunneosetosum*	M35 Consensus 1	FLOR 56395	Brazil	Santa Catarina	MK953366	MK953452	MK944353
*Pterulicium (Pterula) caricispendulae*	KM155784	K(M) 155784	UK	England	MK953367	–	–
*Pterulicium (Pterula)* sp.	F20	INPA 280129	Brazil	Amazonas	MK953370	MK953454	–
*Pterulicium (Pterula)* sp*.*	F21	INPA 280132	Brazil	Amazonas	MK953371	MK953455	MK944355
*Pterulicium (Pterula)* sp.	F26	not deposited	Brazil	Espírito Santo	MK953372	MK953456	MK944356
*Pterulicium (Pterula)* sp.	F30	not deposited	Brazil	Espírito Santo	MK953373	MK953457	MK944357
*Pterulicium (Pterula)* sp*.*	F57	HSTM-Fungos 9925	Brazil	Pará	MK953376	MK953460	MK944359
*Pterulicium (Pterula)* sp*.*	F76 Consensus 1	HSTM-Fungos 9950	Brazil	Pará	MK953382	MK953461	MK944360
*Pterulicium (Pterula)* sp*.*	M1	FLOR 56364	Brazil	Santa Catarina	MK953383	MK953462	MK944361
*Pterulicium (Pterula)* sp*.*	M6	FLOR 56369	Brazil	Santa Catarina	MK953384	MK953463	–
*Pterulicium (Pterulicium) xylogenum*	KM167222	K(M) 167222	Bangladesh	–	MK953387	–	–
*Aphanobasidium pseudotsugae*	K6	K(M) 170662	UK	England	MK953243	MK953402	–
*Aphanobasidium pseudotsugae*	K7	K(M) 180787	UK	Scotland	MK953244	–	–
*Radulomyces confluens*	KM167249	K(M) 167249	Brazil	–	MK953388	–	–
*Radulomyces confluens*	KM167250	K(M) 167250	Argentina	–	MK953389	–	–
*Radulomyces confluens*	KM181613	K(M) 181613	UK	England	MK953390	MK953401	MK944374
*Radulomyces copelandii*	M150	K(M) 173275	USA	–	MK953391	MK953465	–

### Phylogenetic analyses

A preliminary maximum-likelihood (ML) analysis was conducted with the sequences generated in this study alongside GenBank sequences to find the best outgroup for *Pterulaceae* based on previous studies (Dentinger et al. 2016; Zhao et al. 2016; Matheny et al. 2006; Larsson 2007) and to assess the similarities between the cloned sequences (Additional file 1; Additional file 2).

A reduced version of the previous dataset with only one sequence from each cloned sample was created. After removing near-identical sequences with no phylogenetic resolution, the final dataset comprised 119 sequences, including 32 sequences from GenBank and four sequences of *Stephanospora* as outgroups, and was divided into five partitions for further analyses: ITS1, 5.8S, ITS2, LSU and RPB2. Each partition was aligned separately with MAFFT v7.311 (Katoh and Standley 2013) using the E-INS-i algorithm for ITS1 and ITS2, and L-INS-i for 5.8S, LSU and RPB2. The alignments were examined and corrected manually in AliView v1.5 (Larsson 2014) and trimmed to remove uneven ends. Following the simple indel coding (Simmons and Ochoterena 2000), a morphological matrix were constructed using SeqState (Müller 2005) where indels were coded as binary characters. The nucleotide alignments were then trimmed with trimAl v1.4.rev22 (Capella-Gutiérrez et al. 2009) with the option -gappyout to remove unaligned regions.

Maximum-likelihood tree reconstruction was performed with IQ-TREE v1.6.7.1 (Nguyen et al. 2015). The best-fit evolutionary models and partitioning scheme for this analysis were estimated by the built-in ModelFinder (option -m MF + MERGE) allowing the partitions to share the same set of branch lengths but with their own evolution rate (−spp option) (Chernomor et al. 2016; Kalyaanamoorthy et al. 2017). Branch support was assessed with 1000 replicates of ultrafast bootstrapping (UFBoot) (Hoang et al. 2018) and allowing resampling partitions and then sites within these partitions to reduce the likelihood of false positives on branch support (option -bspec GENESITE).

Bayesian Inference (BI) was implemented using MRBAYES v3.2 (Ronquist et al. 2012) with two independent runs, each one with four chains and starting from random trees. The best-fit evolutionary models and partitioning scheme for these analyses were estimated as for the ML analysis but restricting the search to models implemented on MRBAYES (options -m TESTMERGEONLY -mset mrbayes). Chains were run for 10^7^ generations with tree sampling every 1000 generations. The burn-in was set to 25% and the remaining trees were used to calculate a 50% majority consensus tree and Bayesian Posterior Probability (BPP). The convergence of the runs was assessed on TRACER v1.7 (Rambaut et al. 2018) to ensure the potential scale reduction factors (PSRF) neared 1.0 and the effective sample size values (ESS) were sufficiently large (> 200). Nodes with BPP ≥0.95 and/or UFBoot ≥95 were considered strongly supported. Alignment and phylogenetic trees are deposited in Treebase (ID: 24428).

## RESULTS

From this section, all taxa are referred to by the names proposed in this study.

### Field data

Fieldwork resulted in the discovery of approximately 100 new specimens, now placed within *Pterulaceae* (Table 2). Axenic culture isolation was also possible from several of these specimens.

### Phylogenetic analyses

A total of 278 sequences from 123 samples were generated in this study: 153 nrITS, 74 nrLSU and 51 RPB2; 61 from cloning and 40 from genome mining. The final alignment consisted of 113 sequences with 2737 characters and 1050 parsimony-informative sites. The BI analysis converged both runs as indicated by the effective sample sizes (ESS) of all parameters above 2800 and the potential scale reduction factors (PSRF) equal 1.000 for all the parameters according to the 95% HPD Interval.

**Fig. 3:**
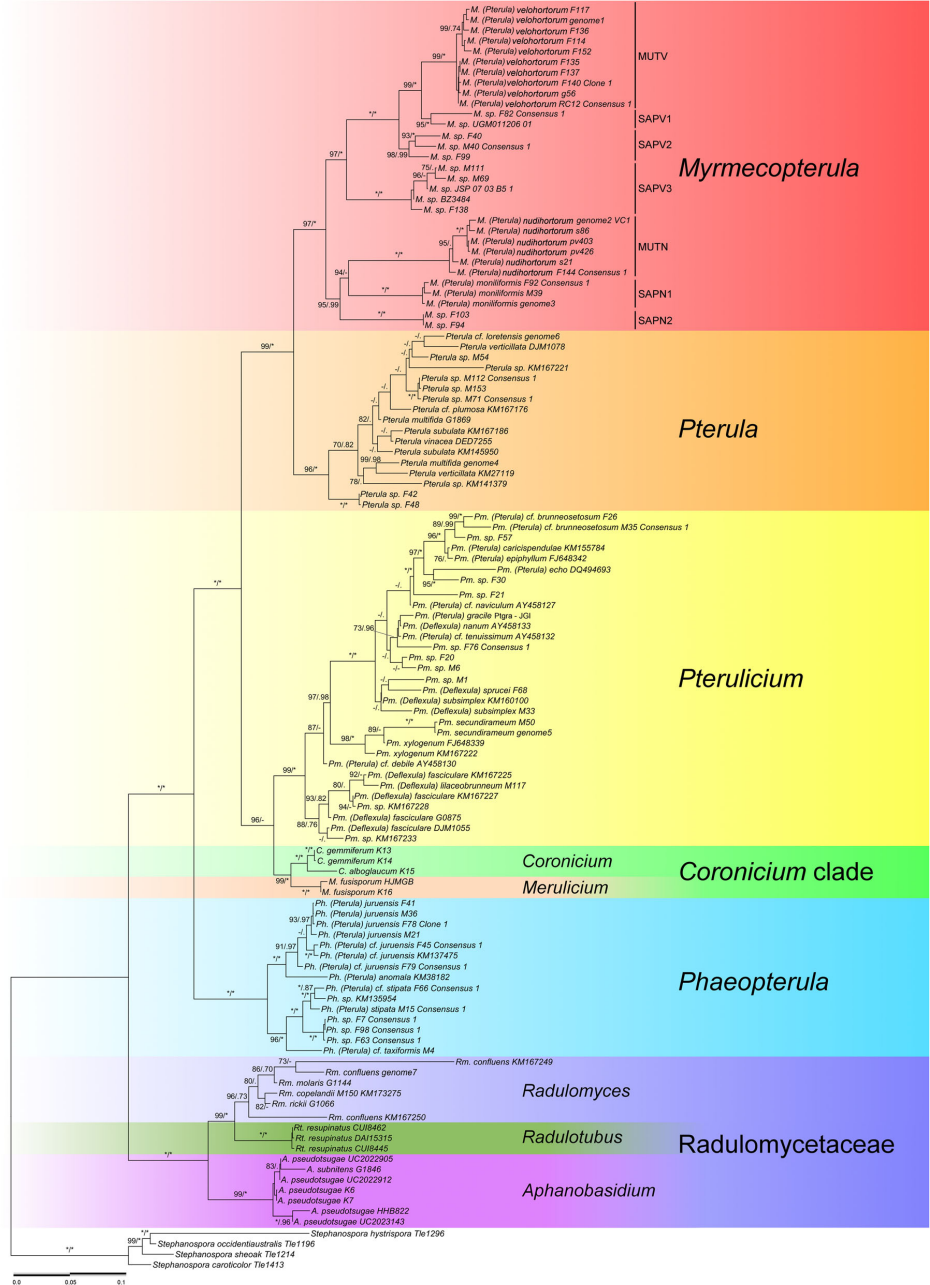
Maximum-likelihood tree of *Pterulaceae* and *Radulomycetaceae*. Support values on the branches are UFBoot/BPP and shown only for UFBoot≥70 and BPP ≥ 0.70 and branch length ≥ 0.003 substitutions per site. Asterisks (*) represent maximum UFBoot/BPP values, dashes (−) represent values below the cut-off threshold (70%), and dots (.) represent ML clades that were not recovered in the BI tree. Details for the complete tree can be found in Additional file 2 and TreeBase (ID: 24428). Scale bar: nucleotide substitutions per site

The new classification proposed in this study (Fig. 3), highlights six main clades containing nine genera: *Radulomycetaceae* (containing *Aphanobasidium*, *Radulotubus* and *Radulomyces*), *Phaeopterula* (hereinafter abbreviated as *Ph*.; previously *Pterula* spp.), *Coronicium* superclade (grouping *Merulicium* and *Coronicium*), *Pterulicium* (previously *Pterulicium*, *Pterula* spp. and *Deflexula* spp.), *Pterula* and *Myrmecopterula* (*Myrmecopterula* gen. nov., previously *Pterula* spp.).

#### Radulomycetaceae (UFBoot = 99; BPP = 1)

This clade groups with strong support three of the five resupinate genera recognized in *Pterulaceae*, namely *Aphanobasidium (UFBoot = 100; BPP = 1), Radulotubus (UFBoot = 100; BPP = 1)* and *Radulomyces (UFBoot = 100; BPP = 0.86).* The placement of *Aphanobasidium* and *Radulomyces* into *Pterulaceae* was previously shown by phylogenetic reconstructions of corticioid taxa (Larsson et al. 2004; Larsson 2007). *Radulotubus* was proposed by Zhao et al. (2016) as sister clade of *Radulomyces* to accommodate one species bearing poroid hymenophore. In our analyses, *Radulotubus* was recovered in the same position as in the original publication. This is the only poroid species within *Pterulaceae*.

No members of the three genera within this superclade are pteruloid (i.e. coralloid basidiomes with dimitic hyphal system) in their morphology and consequently we introduce the family name *Radulomycetaceae* fam. nov. to accommodate them, as discussed further below. The current sister clade to *Pterulaceae* in our analyses is *Stephanosporaceae*, from which members of the *Radulomycetaceae* clade are clearly distinct phylogenetically and morphologically.

#### Phaeopterula (UFBoot = 100; BPP = 1)

*Phaeopterula* received maximum support in both analyses. It includes *Pterula stipata*, *Pt. anomala*, *Pt. juruensis* and other species which all have dark brown basidiomes. This clade is the first coralloid lineage to diverge within *Pterulaceae*. As these species render *Pterula* paraphyletic, a reclassification is needed. The generic name *Phaeopterula* was originally proposed as a subgenus of *Pterula* to accommodate *Ph. hirsuta* and *Ph. juruensis* (Hennings 1900; Hennings 1904). We propose its reintroduction below to distinguish these brown-pigmented taxa from *Pterula* s. str.

#### Coronicium superclade (UFBoot = 98; BPP = 1)

This clade groups the remaining two resupinate genera of *Pterulaceae*, the monospecific *Merulicium* and *Coronicium (UFBoot = 100; BPP = 1)*. Both genera form resupinate basidiomes but differ in the hyphal system present (dimitic in *Merulicium*, monomitic in *Coronicium*). Some *Pterulicium* species also show transitions in their morphology to a resupinate state. Corner (1950) showed that *Pm. xylogenum* Corner could form monomitic corticioid patches independent of the coralloid state and even in its absence, thus appearing to be truly corticioid. Furthermore, experimental studies on *Pm. echo* show a dimitic, resupinate, fertile corticioid phase both on agar and when cultured on cocoa twigs (McLaughlin and McLaughlin 1972; McLaughlin et al. 1978; McLaughlin and McLaughlin 1980). Despite the morphological distinctiveness from the rest of *Pterulaceae*, there is a trend in the morphology and strong phylogeny support for the placement of the *Coronicium* superclade among *Pterula*/*Myrmecopterula* and *Pterulicium* clades within *Pterulaceae*.

#### Pterulicium (UFBoot = 99; BPP = 1)

Two type species, *Pterulicium xylogenum* and *Deflexula fascicularis*, are nested within this clade alongside several species currently assigned to *Pterula* but which all have simple basidiomes (unbranched or limited branching). The *Pterula* species are interspersed with some *Deflexula*, rendering both genera polyphyletic. *Pterulicium xylogenum* forms a well-supported subclade with *Pterula secundiramea* (= *Pt. palmicola*). *Deflexula fascicularis* forms a subclade with other *Deflexula* species that share globose spores, an unusual feature within *Pterulaceae*, most of which form ellipsoid to subamygdaliform spores.

#### Pterula (UFBoot = 100; BPP = 1)

This clade groups the true *Pterula* spp. that are represented by very bushy coralloid basidiomes, usually robust and taller than those of *Pterulicium*, stipe concolorous with hymenophore and lacking a cottony subiculum. *Pterula* has a mainly pantropical and pan-subtropical distribution, with occurrence reported to all continents except Antarctica (Corner 1970).

#### Myrmecopterula (UFBoot = 97; BPP = 1)

This sister clade of *Pterula* represents the newly proposed genus (see below). It groups the two species cultivated by attine ants in the *Apterostigma pilosum* group with *M. moniliformis* and several unidentified free-living species. The species in this clade are only known from the Neotropics. *Myrmecopterula* is divided into seven subclades (Fig. 3) representing the two mutualists (MUTV and MUTN), three closely related to *M. velohortorum* (SAPV 1–3) and two closely related to *M. nudihortorum* (SAPN 1–2).

## TAXONOMY

**Radulomycetaceae** Leal-Dutra, Dentinger, G.W. Griff., **fam. nov.**

MycoBank MB831047.

*Diagnosis:* Differs from resupinate forms of *Pterulaceae* in the monomitic hyphal system and the absence of cystidia. Cystidia may be either present or absent in *Pm. xylogenum*, in the latter case the amygdaliform spores differentiate the species from *Radulomyces* that has ellipsoid to globose spores.

*Etymology*: From the type genus *Radulomyces*.

*Type genus*: *Radulomyces* M.P. Christ. 1960.

*Description*: *Basidiome* resupinate, effused, mostly adnate, ceraceous, hymenophore smooth, tuberculate, odontioid, raduloid or poroid. *Hyphal system* monomitic, generative hyphae with clamps, hyaline, thin- to slightly thick-walled. *Cystidia* absent. *Basidia* terminal clavate or other form if pleural, usually with 4-sterigmata and a basal clamp. *Basidiospores* ellipsoid to globose, hyaline, mostly smooth, thin- to slightly thick-walled, acyanophilous, inamyloid and non-dextrinoid.

*Notes*: *Radulomyces*, *Aphanobasidium* and *Radulotubus* are placed in *Radulomycetaceae*. Larsson (2007) suggested that *Lepidomyces* had affinities to *Aphanobasidium* and could possibly be placed in *Pterulaceae*. However, no sequence data for the genus are available. *Lepidomyces* is described as bearing pleurobasidia as in *Aphanobasidium*, but also leptocystidia as in *Coronicium* and *Merulicium*. Given its morphological similarities to *Aphanobasidium* and the *Coronicium* superclade, we retain *Lepidomyces* as *incertae sedis* until molecular data are available to confirm its phylogenetic position

**Myrmecopterula** Leal-Dutra, Dentinger & G.W. Griff., **gen. nov.**

MycoBank MB831048.

*Etymology*: From the ancient Greek μύρμηκος (=*mýrmēkos*), genitive form of μύρμηξ (=*mýrmēx*), ants. Thus, *Pterula* of the ants, due to the observed relationship of several taxa in this genus with nests of fungus-growing ants.

*Diagnosis:* Differs from *Pterula* in the presence of the cottony subiculum.

*Type species*: *Myrmecopterula moniliformis* (Henn.) Leal-Dutra et al. 2019.

*Description: Basidiome* if present bushy, pteruloid, white-cream to light brown and greyish surface, normally concolorous or stipe with a darker tone than the hymenophore, arising from cottony subiculum with mycelial cords. *Stipe* surface sterile. *Hyphal system*, dimitic hyphal system. *Basidiospores* relatively small spores, usually less than 7 μm wide.

*Ecology:* Usually associated with the nests of ants, growing on top, or from a living or dead nest, or being cultivated by the ants.

*Notes*: Basidiomes of *Myrmecopterula* species are very similar to those of *Pterula* in habit, shape, and colour, but differ in the presence of mycelial cords and a cottony subiculum from which the basidiomes emerge. Some species of *Myrmecopterula* arise from soil, while others superficially appear to grow on wood. Closer observation of basidiomes formed on wood revealed that, rather than being lignicolous, they instead grow from a loose, granular substrate within a cavity inside the wood. This substrate in some cases resembles the substrate in the fungus gardens of the *Apterostigma pilosum* group of ants. In addition, *M. moniliformis*, which arises from soil, has been found emerging from active and inactive attine nests, (S. Sourell, pers. comm.; M. C. Aime, pers. comm.). Thus, all but one of the *Myrmecopterula* clades found to date had some association with attine ants, of which the two farmed mutualist species (*M. nudihortorum* and *M. velohortorum*) are best known. The five other species (of which only *M. moniliformis* is named) are less well studied and may play a role in decomposition of residual substrates in abandoned fungus garden, or potentially even as mycoparasites of the ant cultivar. In contrast, no *Pterula* spp. have any reported association with ants, but instead are found growing directly from wood and leaf litter.

**Myrmecopterula moniliformis** (Henn.) Leal-Dutra, Dentinger & G.W. Griff., **comb. nov.**

MycoBank MB831049.

*Basionym*: *Lachnocladium moniliforme* Henn., *Hedwigia* **43**: 198 (1904).

*Synonyms*: *Pterula moniliformis* (Henn.) Corner, *Ann. Bot.*, *Lond*., *n.s.* **16**: 569 (1952).

*Thelephora clavarioides* Torrend, *Brotéria*, *sér. Bot*. **12**: 61 (1914).

*Description*: Corner (1952b: 546–548).

**Myrmecopterula nudihortorum** (Dentinger) Leal-Dutra, Dentinger & G.W. Griff., **comb. nov.**

MycoBank MB831050.

*Basionym*: *Pterula nudihortorum* Dentinger, *Index Fungorum* **98**: 1 (2014); as *‘nudihortus’*, and later ‘*nudihorta’*.

*Diagnosis*: In the field, recognized by the absence of any veil on the fungus garden in *Apterostigma* nests, usually inside decomposing trunks or underground. In culture, it forms very little aerial mycelium and exhibits very slow growth (2–3 mm/week radial growth rate on PDA at 25C). Hyphal clamps abundant.

*Notes****:*** This species was formerly known as the ant cultivar G4. It is only known from the nest of fungus-growing ants in the *Apterostigma pilosum* group in the *A. manni* subclade (Schultz 2007).

**Myrmecopterula velohortorum** (Dentinger) Leal-Dutra, Dentinger & G.W. Griff., **comb. nov.**

MycoBank MB831051.

*Basionym: Pterula velohortorum* Dentinger. *Index Fungorum* **98**: 1 (2014); as *‘velohortus’*, and later ‘*velohorta’*.

*Diagnosis*: In the field, recognized by the *Apterostigma* garden covered by a mycelial veil, usually inside decomposing trunks, below the leaf litter or hanging on exposed surfaces aboveground. In culture, it forms very cottony aerial mycelia with presence of racquet hyphae (Fig. 5 in Additional file 3). Large and abundant hyphal clamps. Slow growth rate, but faster than *M. nudihortorum*.

*Notes*: This species was formerly known as the ant cultivar G2. It is only known from the nest of fungus-growing ants in the *Apterostigma pilosum* group in the *A. dentigerum* subclade (Schultz 2007).

**Phaeopterula** (Henn.) Sacc. & D. Sacc., *Syll. Fung.* **17**: 201 (1905).

*Basionym*: *Pterula* subgen. *Phaeopterula* Henn., *Monsunia* **1**: 9 (1900) [“1899”].

*Type species*: *Phaeopterula hirsuta* (Henn.) Sacc. & D. Sacc. 1899.

*Description*: *Basidiomes* pteruloid, solitary or gregarious, scarcely branched to almost bushy, monopodial and slightly symmetric, branches from light brownish pink or greyish to pale brown and stipe dark reddish to rusty brown. *Stipe* surface glabrous with agglutinated hyphae (not sclerotioid) to villose-tomentose. Dark brown mycelial cords usually present. *Hyphal system* dimitic with thick-walled skeletal hyphae, generative hyphae thin-walled and often clamped. *Hymenial cystidia* absent, caulocystidia sometimes present. *Basidia* terminal, clavate to suburniform. *Basidiospores* less than 9 μm varying between pip-shaped, subamygdaliform and ellipsoid.

*Ecology*: Growing on dead twigs or dead wood.

*Notes*: Hennings (1900) recognized the subgenus *Phaeopterula* to accommodate *Pterula hirsuta* that was distinguished from other *Pterula* species by the reportedly brown spores. Hennings (1904) later described a second species in the subgenus, *Ph. juruensis*, but noted that it was morphologically quite distinct from *Ph. hirsuta*. *Phaeopterula* was raised to generic level by Saccardo and Saccardo (1905) who cited only *Ph. juruensis*. *Pterula hirsuta* was recombined in *Dendrocladium* by Lloyd (1919) but later returned to *Pterula* by Corner (1950), even though Corner did not confirm the presence of brown spores in the samples he examined. Although we also have not observed pigmented spores in any of these taxa, dark brown pigments in the stipe hyphae are a consistent and diagnostic feature in this group, so we resurrect the name *Phaeopterula*. The term ‘*Phaeo-’* relates to brown-pigmented basidiospores, but while members of this genus do not have brown basidiospores, they do contain brown hyphal pigments.

**Phaeopterula anomala** (P. Roberts) Leal-Dutra, Dentinger, G.W. Griff., **comb. nov.**

MycoBank MB830999.

*Basionym*: *Pterula anomala* P. Roberts, *Kew Bull.* **54**(3): 528 (1999).

*Description*: Roberts (1999: 528–529).

**Phaeopterula hirsuta** (Henn.) Sacc. & D. Sacc., *Syll. fung.* (Abellini) **17**: 201 (1905).

MycoBank MB469044.

*Basionym*: *Pterula hirsuta* Henn., *Monsunia* **1**: 9 (1899) [1900].

*Synonym*: *Dendrocladium hirsutum* (Henn.) Lloyd, *Mycol. Writ*. **5**: 870 (1919).

*Description*: Corner (1950: 517).

**Phaeopterula juruensis** Henn. ex Sacc. & D. Sacc., *Syll. Fung.* **17**: 201 (1905).

MycoBank MB634235.

*Basionym:* *Phaeopterula juruensis* Henn. ex Sacc. & D. Sacc., *Syll. Fung.* 17: 201 (1905).

*Synonym*: *Dendrocladium juruense* (Henn. ex Sacc. & D. Sacc.) Lloyd, *Mycol. Writ*. **5**: 870 (1919).

*Pterula juruensis* (Henn. ex Sacc. & D. Sacc.) Corner, *Monogr. Clavaria.*: 518 (1950).

*Phaeopterula juruensis* Henn., Hedwigia 43 (3): 175 (1904).

*Descriptions*: Corner (1950:518; 1952b: 542–544).

***Phaeopterula stipata*** (Corner) Leal-Dutra, Dentinger, G.W. Griff., **comb. nov.**

MycoBank MB831000.

*Basionym*: *Pterula stipata* Corner, *Ann. Bot*., Lond., n.s. **16**: 568 (1952).

*Description*: Corner (1952b: 556–557).

**Phaeopterula taxiformis** (Mont.) Leal-Dutra, Dentinger, G.W. Griff., **comb. nov.**

MycoBank MB831001.

*Basionym*: *Pterula taxiformis* Mont., *Syll. Gen.*: 181 (1856).

*Synonyms*: *Lachnocladium taxiforme* (Mont.) Sacc., *Syll. Fung.* **6**: 740 (1888).

*Pterula humilis* Speg., *Revista Argent. Hist. Nat*. **1**(2): 110 (1891).

*Pterula humilis* var. *tucumanensis* Speg., *Anal. Mus. nac. B. Aires*, Ser. 3 **12**: 280 (1909).

*Descriptions*: Corner (1950: 523–524; 1952b: 560–561).

**Phaeopterula taxiformis var. gracilis** (Corner) Leal-Dutra, Dentinger, G.W. Griff., **comb. nov.**

MycoBank MB831002.

*Basionym*: *Pterula taxiformis* var. *gracilis* Corner, *Ann. Bot.*, Lond., n.s. **16**: 568 (1952).

*Description*: Corner (1952b: 561).

**Pterulicium** Corner, *Monogr. Clavaria.*: 699 (1950).

*Synonym: Deflexula* Corner, *Monogr. Clavaria.*: 695 (1950).

*Type Species*: *Pterulicium xylogenum* (Berk. & Broome) Corner 1950.

*Description*: *Basidiomes* pteruloid rarely corticioid, solitary or gregarious, simple or scarcely branched, occasionally exhibiting abundant unilateral branching (Figs. 1i, l), varying from creamy white to brown on the stipe and creamy white on the tips or creamy white or pale lilaceous to pale brown on uniformly coloured basidiomes. *Stipe* surface sometimes sclerotioid (see Corner 1950). *Hyphal system* dimitic with slightly thick-walled skeletal hyphae, generative hyphae thin-walled and often clamped. *Hymenial cystidia* usually present, caulocystidia sometimes present. Basidia terminal, clavate to suburniform. *Basidiospores* shape varying between globose to subglobose, pip-shaped, amygdaliform to subamygdaliform, ellipsoid.

*Ecology*: On dead leaves, dead twigs or dead wood, rarely as a pathogen or endophyte of living plants.

*Notes*: *Deflexula* is synonymised with *Pterulicium* in this study. In addition, several species previously placed in *Pterula* are transferred to *Pterulicium* below. Other *Pterula* species that might need to be recombined in *Pterulicium*, require further investigation since their original descriptions do not provide enough information to confidently assign them here.

**Pterulicium argentinum** (Speg.) Leal-Dutra, Dentinger, G.W. Griff., **comb. nov.**

MycoBank MB831003.

*Basionym*: *Mucronella argentina* Speg., *Anal. Mus. nac. Hist. nat. B. Aires* **6**: 178 (1899) [“1898”].

*Synonyms*: *Deflexula argentina* (Speg.) Corner, *Ann. Bot.*, Lond., n.s. **16**: 276 (1952).

*Deflexula lilaceobrunnea* var. *elongata* Corner, *Ann. Bot*., Lond., n.s. **16**: 276 (1952).

*Descriptions*: Corner (1952a: 276; 1970: 196).

**Pterulicium argentinum var. ramosum** (Corner) Leal-Dutra, Dentinger, G.W. Griff., **comb. nov.**

MycoBank MB831004.

*Basionym*: *Deflexula argentina* (Speg.) Corner, *Ann. Bot*., Lond., n.s. **16**: 276 (1952).

*Description*: Corner (1970: 197).

**Pterulicium bambusae** (Corner) Leal-Dutra, Dentinger, G.W. Griff., **comb. nov.**

MycoBank MB831005.

*Basionym*: *Pterula bambusae* Corner, *Beih. Nova Hedwigia* **33**: 209 (1970).

*Description*: Corner (1970: 209).

**Pterulicium bromeliphilum** (Corner) Leal-Dutra, Dentinger, G.W. Griff., **comb. nov.**

MycoBank MB831006.

*Basionym*: *Pterula bromeliphil*a Corner, *Beih. Nova Hedwigia* **33**: 210 (1970)

*Description*: Corner (1970: 210).

**Pterulicium brunneosetosum** (Corner) Leal-Dutra, Dentinger, G.W. Griff., **comb. nov.**

MycoBank MB831007.

*Basionym*: *Pterula brunneosetosa* Corner, *Ann. Bot*., Lond., n.s. **16**: 566 (1952).

*Descriptions*: Corner (1952b: 535–536; 1970: 210).

**Pterulicium campoi** (Speg.) Leal-Dutra, Dentinger, G.W. Griff., **comb. nov.**

MycoBank MB831008.

*Basionym*: *Pterula campoi* Speg., *Bol. Acad. nac. Cienc. Córdoba* **25**: 29 (1921).

*Descriptions*: Corner (1970: 210–211) and Spegazzini (1921: 29–30).

**Pterulicium caricis-pendulae** (Corner) Leal-Dutra, Dentinger, G.W. Griff., **comb. nov.**

MycoBank MB831009.

*Basionym*: *Pterula caricis-pendulae* Corner, *Beih. Nova Hedwigia* **33**: 211 (1970).

*Description*: Corner (1970: 211–212).

**Pterulicium crassisporum** (P. Roberts) Leal-Dutra, Dentinger, G.W. Griff., **comb. nov.**

MycoBank MB831010.

*Basionym*: *Pterula crassispora* P. Roberts, *Kew Bull*. **54**: 531 (1999).

*Description*: Roberts (1999: 531–532).

**Pterulicium cystidiatum** (Corner) Leal-Dutra, Dentinger, G.W. Griff., **comb. nov.**

MycoBank MB831011.

*Basionym*: *Pterula cystidiata* Corner, *Ann. Bot*., Lond., n.s. **16**: 567 (1952).

*Description*: Corner (1952b: 537–539).

**Pterulicium debile** (Corner) Leal-Dutra, Dentinger, G.W. Griff., **comb. nov.**

MycoBank MB831012.

*Basionym*: *Pterula bromeliphil*a Corner, *Monogr. Clavaria.*: 698 (1950).

*Description*: Corner (1950: 508–510).

**Pterulicium echo** (D.J. McLaughlin & E.G. McLaughlin) Leal-Dutra, Dentinger, G.W. Griff., **comb. nov.**

MycoBank MB831013.

*Basionym*: *Pterula echo* D.J. McLaughlin & E.G. McLaughlin, *Can. J. Bot*. **58**: 1328 (1980).

*Description:* McLaughlin and McLaughlin (1980: 1328–1332).

**Pterulicium epiphylloides** (Corner) Leal-Dutra, Dentinger, G.W. Griff., **comb. nov.**

MycoBank MB831014.

*Basionym*: *Pterula epiphylloides* Corner, *Ann. Bot*., Lond., n.s. **16**: 567 (1952).

*Description*: Corner (1952b: 540).

**Pterulicium epiphyllum** (Corner) Leal-Dutra, Dentinger, G.W. Griff., **comb. nov.**

MycoBank MB831015.

*Basionym: Pterula epiphylla* Corner *Monogr. Clavaria.*: 698 (1950).

*Description:* Corner (1950: 510–511).

**Pterulicium fasciculare** (Bres. & Pat.) Leal-Dutra, Dentinger, G.W. Griff., **comb. nov.**

MycoBank MB831016.

*Basionym: Pterula fascicularis* Bres. & Pat., *Mycol. Writ*. **1**: 50 (1901).

*Synonym*: *Deflexula fascicularis* (Bres. & Pat.) Corner, *Monogr. Clavaria.*: 395 (1950).

*Description:* Corner (1950: 395–397).

**Pterulicium fluminense** (Corner) Leal-Dutra, Dentinger, G.W. Griff., **comb. nov.**

MycoBank MB831017.

*Basionym: Pterula fluminensis* Corner, *Ann. Bot.*, Lond., n.s. **16**: 567 (1952).

*Descriptions:* Corner (1952b: 541–542; 1970: 215).

**Pterulicium gordium** (Speg.) Leal-Dutra, Dentinger, G.W. Griff., **comb. nov.**

MycoBank MB831018.

*Basionym: Clavaria gordius* Speg., *Anal. Soc. cient. Argent*. **17**(2): 83 (1884).

*Synonym*: *Pterula gordius* (Speg.) Corner, *Monogr. Clavaria.*: 513 (1950).

*Description:* Corner (1950: 513–514).

**Pterulicium gordium var. macrosporum** (Corner) Leal-Dutra, Dentinger, G.W. Griff., **comb. nov.**

MycoBank MB831019.

*Basionym: Pterula gordius* var. *macrospora* Corner, *Proc. Linn. Soc*. London **178**: 100 (1967).

*Description:* Corner (1967: 100–101).

**Pterulicium gracile** (Desm. & Berk.) Leal-Dutra, Dentinger, G.W. Griff., **comb. nov.**

MycoBank MB831020.

*Basionym: Typhula gracilis* Desm. & Berk., Ann. nat. Hist., Mag. Zool. Bot. Geol. 1: 202 (1838).

*Synonyms*: *Pistillaria gracilis* (Desm. & Berk.) Pat., *Tab. analyt. Fung*. (Paris)(6): 30 (1886).

*Hirsutella gracilis* (Desm. & Berk.) Pat., *Revue mycol.*, Toulouse **14**(no. 54): 69 (1892).

*Pterula gracilis* (Desm. & Berk.) Corner, *Monogr. Clavaria.*: 514 (1950).

*Clavaria aculina* Quél., *C. r. Assoc. Franç. Avancem. Sci*. **9**: 670 (1881) [1880].

*Pistillaria aculina* (Quél.) Pat., *Tab. analyt. Fung.* (Paris)(6): 29 (Fig. 570) (1886).

*Ceratella aculina* (Quél.) Pat., *Hyménomyc. Eur*. (Paris): 157 (1887).

*Cnazonaria aculina* (Quél.) Donk, *Meded. Bot. Mus. Herb. Rijks Univ*. Utrecht **9**: 97 (1933).

*Pistillaria aculina* subsp. *juncicola* Bourdot & Galzin, *Hyménomyc. de France* (Sceaux): 138 (1928) [1927].

*Pistillaria aculina* subsp. *graminicola* Bourdot & Galzin, *Hyménomyc. de France* (Sceaux): 139 (1928) [1927].

*Pistillaria aculina* subsp. *acicula* Bourdot & Galzin, *Hyménomyc. de France* (Sceaux): 139 (1928) [1927].

*Typhula brunaudii* Quél., *C. r. Assoc. Franç. Avancem. Sci*. **13**: 283 (1885) [1884].

*Clavaria brunaudii* (Quél.) Sacc., *Syll. fung*. (Abellini) **6**: 730 (1888).

*Ceratella ferryi* Quél. & Fautrey, *Revue mycol.*, Toulouse **15**(no. 57): 15 (1893).

*Pistillaria ferryi* (Quél. & Fautrey) Sacc., *Syll. fung*. (Abellini) **11**: 141 (1895).

*Pistillaria ferryi* subsp. *tremula* Sacc., *Syll. fung*. (Abellini) **17**: 202 (1905).

*Mucronella rickii* Oudem., *Ned. kruidk. Archf*, 3 sér. **2**(3): 667 (1902).

*Cnazonaria rickii* (Oudem.) Donk, *Meded. Bot. Mus. Herb. Rijks Univ*. Utrecht **9**: 99 (1933).

*Ceratellopsis rickii* (Oudem.) Corner, *Monogr. Clavaria.*: 205 (1950).

*Description:* Corner (1950: 514–516).

**Pterulicium incarnatum** (Pat.) Leal-Dutra, Dentinger, G.W. Griff., **comb. nov.**

MycoBank MB831021.

*Basionym: Pterula incarnata* Pat., in Patouillard & Lagerheim, *Bull. Herb. Boissier* **3**(1): 58 (1895).

*Descriptions:* Corner (1950: 517; 1970: 215–216).

**Pterulicium intermedium** (Dogma) Leal-Dutra, Dentinger, G.W. Griff., **comb. nov.**

MycoBank MB831022.

*Basionym: Pterula intermedia* Dogma, *Philipp. Agric*. **49**: 852 (1966).

*Descriptions:* Corner (1970): 216 and Dogma (1966: 852-855).

**Pterulicium laxum** (Pat.) Leal-Dutra, Dentinger, G.W. Griff., **comb. nov.**

MycoBank MB831023.

*Basionym: Pterula laxa* Pat., *Bull. Soc. mycol. Fr*. **18**(2): 175 (1902).

*Descriptions:* Corner (1950: 518; 1970: 217).

**Pterulicium lilaceobrunneum** (Corner) Leal-Dutra, Dentinger, G.W. Griff., **comb. nov.**

MycoBank MB831024.

*Basionym: Deflexula lilaceobrunnea* Corner, *Monogr. Clavaria.*: 695 (1950).

*Description:* Corner (1950: 397–398).

**Pterulicium lilaceobrunneum var. evolutius** (Corner) Leal-Dutra, Dentinger, G.W. Griff., **comb. nov.**

MycoBank MB831025.

*Basionym: Deflexula lilaceobrunnea* var. *evolutior* Corner, *Beih. Nova Hedwigia* **33**: 197 (1970).

*Description:* Corner (1970: 197–198).

**Pterulicium longisporum** (Corner) Leal-Dutra, Dentinger, G.W. Griff., **comb. nov.**

MycoBank MB831026.

*Basionym: Pterula longispora* Corner, *Ann. Bot*., Lond., n.s. **16**: 567 (1952).

*Description:* Corner (1952b: 544–545).

**Pterulicium macrosporum** (Pat.) Leal-Dutra, Dentinger, G.W. Griff., **comb. nov.**

MycoBank MB831027.

*Basionym: Ceratella macrospora* Pat., in Patouillard & Lagerheim, *Bull. Soc. mycol. Fr*. **8**(3): 119 (1892).

*Synonyms*: *Pistillaria macrospora* (Pat.) Sacc., *Syll. fung*. (Abellini) **11**: 142 (1895).

*Pterula macrospora* (Pat.) Corner, *Monogr. Clavaria.*: 518 (1950).

*Descriptions:* Corner (1950: 518; 1970: 218).

**Pterulicium majus** (Corner) Leal-Dutra, Dentinger, G.W. Griff., **comb. nov.**

MycoBank MB831028.

*Basionym: Deflexula major* Corner, *Ann. Bot*., Lond., n.s. **16**: 277 (1952).

*Description:* Corner (1952a: 277–278).

**Pterulicium mangiforme** (Corner) Leal-Dutra, Dentinger, G.W. Griff., **comb. nov.**

MycoBank MB831029.

*Basionym: Deflexula mangiformis* Corner, *Ann. Bot.*, Lond., n.s. **16**: 278 (1952).

*Description:* Corner (1952a: 278).

**Pterulicium microsporum** (Corner) Leal-Dutra, Dentinger, G.W. Griff., **comb. nov.**

MycoBank MB831030.

*Basionym: Deflexula microspora* Corner, *Bull. Jard. bot. État Brux.* **36**: 264 (1966).

*Description:* Corner (1966: 264).

**Pterulicium nanum** (Pat.) Leal-Dutra, Dentinger, G.W. Griff., **comb. nov.**

MycoBank MB831031.

*Basionym: Pterula nana* Pat., Bull. *Soc. mycol. Fr.* **18**(2): 175 (1902).

*Synonyms*: *Deflexula nana* (Pat.) Corner, *Bull. Jard. bot. État Brux.* **36**: 264 (1966).

*Pterula vanderystii* Henn. [as *‘vanderysti’*], *Ann. Mus. Congo Belge*, Bot., Sér. 5 **2**(2): 96 (1907).

*Deflexula vanderystii* (Henn.) Corner, *Ann. Bot*., Lond., n.s. **16**: 284 (1952).

*Description:* Corner (1966: 264).

**Pterulicium naviculum** (Corner) Leal-Dutra, Dentinger, G.W. Griff., **comb. nov.**

MycoBank MB831032.

*Basionym: Pterula navicula* Corner, *Ann. Bot*., Lond., n.s. **16**: 568 (1952).

*Description:* Corner (1952b: 549–550).

**Pterulicium oryzae** (Remsberg) Leal-Dutra, Dentinger, G.W. Griff., **comb. nov.**

MycoBank MB831033.

*Basionym: Pistillaria oryzae* Remsberg, *Mycologia* **32**(5): 668 (1940).

*Synonym*: *Pterula oryzae* (Remsberg) Corner, *Monogr. Clavaria.*: 519 (1950).

*Descriptions:* Corner (1950: 519–520) and Remsberg (1940: 668–670).

**Pterulicium phyllodicola** (Corner) Leal-Dutra, Dentinger, G.W. Griff., **comb. nov.**

MycoBank MB831034.

*Basionym: Pterula phyllodicola* Corner, *Beih. Nova Hedwigia* **33**: 220 (1970).

*Description:* Corner (1970: 220).

**Pterulicium phyllophilum** (McAlpine) Leal-Dutra, Dentinger, G.W. Griff., **comb. nov.**

MycoBank MB831035.

*Basionym: Clavaria phyllophila* McAlpine, *Agric. Gaz. N.S.W.*, Sydney **7**: 86 (1896).

*Synonym*: *Pterula phyllophila* (McAlpine) Corner, *Monogr. Clavaria.*: 520 (1950).

*Description:* Corner (1950: 520).

**Pterulicium rigidum** (Donk) Leal-Dutra, Dentinger, G.W. Griff., **comb. nov.**

MycoBank MB831036.

*Basionym: Pterula rigida* Donk, *Monogr. Clavaria.*: 698 (1950).

*Description:* Corner (1950: 521).

**Pterulicium sclerotiicola** (Berthier) Leal-Dutra, Dentinger, G.W. Griff., **comb. nov.**

MycoBank MB831037.

*Basionym: Pterula sclerotiicola* Berthier, *Bull. trimest. Soc. mycol. Fr.* **83**: 731 (1968) [1967].

*Description:* Corner (1970: 221).

**Pterulicium secundirameum** (Lév) Leal-Dutra, Dentinger, G.W. Griff., **comb. nov.**

MycoBank MB831038.

*Basionym: Clavaria secundiramea* Lév., *Annls Sci. Nat., Bot*., sér. 3 **2**: 216 (1844).

*Synonyms*: *Pterula secundiramea* (Lév.) Speg., *Bol. Acad. nac. Cienc. Córdoba* **11**(4): 466 (1889).

*Deflexula secundiramea* (Lév.) Corner, *Beih. Nova Hedwigia* **33**: 199 (1970).

*Pterula palmicola* Corner, *Ann. Bot.*, Lond., n.s. **16**: 568 (1952).

*Descriptions:* Corner (1950: 521–522; 1952b: 555–556).

*Notes:* The synonymisation of *Pm. palmicola* (samples M50 and M83) in *Pm. secundirameum* (samples M70 and genome5) is based on our phylogenetic results and morphological comparisons. The only morphological difference between the two species is the shape of the basidiome, however, the other characters are similar and both species are nested together within our tree (Additional file 2).

**Pterulicium sprucei** (Mont.) Leal-Dutra, Dentinger, G.W. Griff., **comb. nov.**

MycoBank MB831039.

*Basionym: Hydnum sprucei* Mont., *Syll. gen. sp. crypt.* (Paris): 173 (1856).

*Synonyms*: *Pterula sprucei* (Mont.) Lloyd, *Mycol. Writ.* **5**: 865 (1919).

*Deflexula sprucei* (Mont.) Maas Geest., *Persoonia* **3**(2): 179 (1964).

*Pterula pennata* Henn., *Hedwigia* **43**(3): 174 (1904).

*Deflexula pennata* (Henn.) Corner, *Ann. Bot.*, Lond., n.s. **16**: 278 (1952).

*Descriptions:* Corner (1952a: 278–279 as ‘*D. pennata’*; 1970: 200–201) and Maas Geesteranus (1964: 178–179).

**Pterulicium subsimplex** (Henn.) Leal-Dutra, Dentinger, G.W. Griff., **comb. nov.**

MycoBank MB831040.

*Basionym: Pterula subsimplex* Henn., *Hedwigia* **36**(4): 197 (1897).

*Synonyms*: *Deflexula subsimplex* (Henn.) Corner, *Ann. Bot.*, Lond., n.s. **16**: 279 (1952).

*Pterula nivea* Pat., *Bull. Soc. mycol. Fr.* **18**(2): 174 (1902).

*Deflexula nivea* (Pat.) Corner, *Monogr. Clavaria.*: 398 (1950).

*Mucronella pacifica* Kobayasi, *Bot. Mag.*, Tokyo **53**: 160 (1939).

*Deflexula pacifica* (Kobayasi) Corner, *Monogr. Clavaria.*: 399 (1950).

*Descriptions:* Corner (1952a: 279–282; 1950: 399 as ‘*D. pacifica*’.

**Pterulicium subsimplex var. multifidum** (Corner) Leal-Dutra, Dentinger, G.W. Griff., **comb. nov.**

MycoBank MB831041.

*Basionym: Deflexula subsimplex* var. *multifida* Corner, *Ann. Bot.*, Lond., n.s. **16**: 282 (1952).

*Description:* Corner (1952a: 282–283).

**Pterulicium subtyphuloides** (Corner) Leal-Dutra, Dentinger, G.W. Griff., **comb. nov.**

MycoBank MB831042.

*Basionym: Pterula subtyphuloides* Corner, *Monogr. Clavaria.*: 698 (1950).

*Description:* Corner (1950: 522–523).

**Pterulicium sulcisporum** (Corner) Leal-Dutra, Dentinger, G.W. Griff., **comb. nov.**

MycoBank MB831043.

*Basionym: Deflexula sulcispora* Corner, *Ann. Bot.*, Lond., n.s. **16**: 283 (1952).

*Description:* Corner (1952a: 283–284).

**Pterulicium tenuissimum** (M.A. Curtis) Leal-Dutra, Dentinger, G.W. Griff., **comb. nov.**

MycoBank MB831044.

*Basionym: Typhula tenuissima* M.A. Curtis, *Am. Journ. Art. Scienc*. **6**: 351 (1848).

*Synonym*: *Pterula tenuissima* (M.A. Curtis) Corner, *Monogr. Clavaria.*: 524 (1950).

*Description:* Corner (1950: 524).

**Pterulicium typhuloides** (Corner) Leal-Dutra, Dentinger, G.W. Griff., **comb. nov.**

MycoBank MB832820.

*Basionym: Pterula typhuloides* Corner, *Monogr. Clavaria.*: 698 (1950).

*Description:* Corner (1950: 525–526).

**Pterulicium typhuloides var. minor** (Corner) Leal-Dutra, Dentinger, G.W. Griff., **comb. nov.**

MycoBank MB832821.

*Basionym: Pterula typhuloides var. minus* Corner, *Monogr. Clavaria.*: 699 (1950).

*Description:* Corner (1950: 526–527).

**Pterulicium ulmi** (Peck) Leal-Dutra, Dentinger, G.W. Griff., **comb. nov.**

MycoBank MB831045.

*Basionym: Mucronella ulmi* Peck, *Ann. Rep. Reg. N.Y. St. Mus*. **54**: 154 (1902) [1901].

*Synonym*: *Deflexula ulmi* (Peck) Corner, *Monogr. Clavaria.*: 400 (1950).

*Descriptions:* Corner (1950: 400; 1970: 202).

**Pterulicium velutipes** (Corner) Leal-Dutra, Dentinger, G.W. Griff., **comb. nov.**

MycoBank MB831046.

*Basionym: Pterula velutipes* Corner, *Ann. Bot.*, Lond., n.s. **16**: 569 (1952).

*Description:* Corner (1952b: 565–566).

**Key to genera of*Pterulaceae*and*Radulomycetaceae***

**Table:** 

1	Cultivated by ants of the *Apterostigma pilosum* group	Myrmecopterula*
	Not cultivated by ants	2
2 (1)	Basidiomes resupinate to effused	3
	Basidiomes coralloid, thread like or allantoid**	10
3 (2)	Hymenophore surface poroid	**Radulotubus**
	Hymenophore surface smooth, tuberculate, odontioid to raduloid or merulioid	4
4 (3)	Cystidia present	5
	Cystidia absent	8
5 (4)	Hyphal system monomitic	6
	Hyphal system dimitic	7
6 (5)	Spores ellipsoid to navicular, thin-walled, cystidia with incrustation	**Coronicium**
	Spores amygdaliform, slightly thick-walled, cystidia smooth	Pterulicium xylogenum***
7 (5)	Hymenophore surface merulioid, presence of cystidia with resinous excretion	**Merulicium**
	Hymenophore surface smooth, cystidia smooth	Pterulicium echo***
8 (4)	Basidia formed laterally from generative hyphae (pleural)	**Aphanobasidium**
	Basidia formed at the end of generative hyphae (terminal)	9
9 (8)	Spores ellipsoid to globose	**Radulomyces**
	Spores amygdaliform	Pterulicium xylogenum***
10 (2)	Basidiome allantoid with swollen fertile regions intercalating with mycelial chords	**Allantula**
	Basidiome coralloid or thread like	11
11 (10)	Stipe and base of branches very dark brown fading towards the tips	**Phaeopterula**
	Basidiomes concolourous or only the stipe light brown coloured	12
12 (11)	Basidiomes simple or scarcely branched, growing up- or downwards	**Pterulicium**
	Basidiomes densely ramified, always ageotropic	13
13 (12)	Cottony subiculum present, associated with attine ants	**Myrmecopterula**
	Cottony subiculum absent, without association with attine ants	**Pterula**

* *Myrmecopterula* cultivated by *Apterostigma* was never reported forming basidiomes

** Allantoid = sausage-shaped, in this case with inflated portions of hymenium intercalating with rhizomorph (see *Allantula* in Corner 1952c)

*** *Pterulicium xylogenum* and *Pm. echo* can have corticioid growth independently of coralloid basidiomes. The cystidia in the former may be either present or absent

## DISCUSSION

### Introduction of *Radulomycetaceae*

We consider that it is better to erect a new family for these three genera (i.e. *Radulomyces*, *Radulotubus* and *Aphanobasidium*) than to leave them in *Pterulaceae* where they are clearly phylogenetically and morphologically distinct from nearly all the other members of *Pterulaceae*. In contrast, *Merulicium* (Fig. 2b-c) and *Coronicium* (Fig. 2a) form corticioid basidiomes but our phylogenetic analyses place them clearly within Pterulaceae. Two *Pterulicium* species, *Pm. echo* and *Pm. xylogenum*, also form both pteruloid and corticioid basidiomes, either independently or together (McLaughlin and McLaughlin 1980; Corner 1950).

Whilst the corticioid basidiomes of *Merulicium* and *Pm. echo* contain a dimitic hyphal system, typical of Pterulaceae, those of *Coronicium* spp. and *Pterulicium xylogenum* form a monomitic hyphal system, like all members of Radulomycetaceae. However, no members of Radulomycetaceae form cystidia, whereas these cells are found in most Pterulaceae (Corner 1950, 1952a, 1952b, 1967, 1970; McLaughlin and McLaughlin 1980; Bernicchia and Gorjón 2010), including *Coronicium* spp. Thus, *Radulomycetaceae* is morphologically characterized by the combination of resupinate basidiomes, monomitic hyphal system and lack of cystidia. Moreover, our phylogenetic analyses strongly support the segregation of *Radulomycetaceae* from *Pterulaceae*.

### Reintroduction of *Phaeopterula*

*Phaeopterula* spp. are distinct from other pterulaceous genera due to the distinctive brown colour of the main axis of the basidiome and monopodial/symmetric branching of these structures. This contrasts with other *Pterulaceae* which are either highly branched (bushy) and of uniform colour (*Pterula* and *Myrmecopterula*) or pigmented only at the stipe base, and (mostly) unbranched (*Pterulicium*). Hennings (1900) originally defined *Phaeopterula* by its brown spores. Corner (1950) cast doubt on the significance of this trait, but our results show that, despite an apparently misguided justification, Hennings was correct to group *Ph. juruensis* with *Ph. hirsuta*.

All *Phaeopterula* spp. are exclusively found on decaying wood, whereas members of other genera of Pterulaceae inhabit more diverse lignocellulosic substrates. Given the basal position of *Phaeopterula* in Pterulaceae, and the fact that all members of the sister family Radulomycetaceae are also lignicolous on wood, this habit is parsimoniously the ancestral condition. The reintroduction of *Phaeopterula* aims to pay tribute to Paul Hennings’ work and his contribution to the taxonomy of Pterulaceae.

### Synonymy of *Deflexula* with *Pterulicium*

Besides the paraphyly represented by *Phaeopterula*, the *Pterulicium* clade shows polyphyly of *Pterula* and *Deflexula*. Several species in the two latter genera are intermixed in a strongly supported subclade (Fig. 3). The presence of the type species of both *Deflexula* and *Pterulicium* within this clade requires that only one name be kept. Both genera were proposed by Corner (1950), to accommodate the dimitic and coralloid (but non-bushy) species, not fitting the description of *Pterula*. The name *Pterulicium* was based on a ‘portmanteau’ combination of *Pterula* and *Corticium* to reflect the presence of a corticioid patch at the stipe base (Corner 1950). However, this patch has only been reported in two species, *Pterulicium xylogenum* (Corner 1950) and *Pm. echo* (McLaughlin and McLaughlin 1980). *Deflexula* was named for the downward-oriented (positively geotropic) basidiomes (Corner 1950). Corner (1950) stated that the resupinate patch in *Pterulicium xylogenum* is monomitic, can exist independently of the coralloid basidiome and is fertile when facing downward; he suggested that there was a close similarity between *Deflexula* and *Pterulicium* in the way the resupinate patch develops from the base of the basidiome. He also made a case for the formation of a fertile hymenium when facing downward in the two genera as supporting this similarity. Nonetheless, experimental studies on *Pm. echo* show that orientation of the hymenium does not affect the ability to produce spores, i.e., the hymenium is ageotropic (McLaughlin et al. 1978) and raised doubts about the validity of the genus *Deflexula*. This morphological distinction is not supported by phylogenetic analysis (Dentinger et al. 2009, Fig. 3) and its emphasis through taxonomic preservation would perpetuate misunderstanding. Accordingly, we propose to retain *Pterulicium* for this clade to avoid major misinterpretations of the species morphology.

### Introduction of *Myrmecopterula* gen. nov.

Two species of Pterulaceae are cultivated by fungus-farming ants of the *Apterostigma pilosum* group in South and Central America (Dentinger et al. 2009; Munkacsi et al. 2004; Villesen et al. 2004; Mueller et al. 2018). Despite intensive investigation, neither has been observed to form basidiomes, but *M. velohortorum* is characterised by the formation of a veil of mycelium around the fungus garden, whilst *M. nudihortorum* lacks this veil. We recovered both species in a strongly supported clade, as a sister clade of *Pterula*, alongside five other subclades containing fertile, apparently free-living species.

All the samples in this clade were collected from neotropical habitats (Fig. 1a-f), mostly as part of our recent fieldwork. During sampling campaigns by ourselves and others, it was observed that many of the ‘free-living’ specimens were associated in some way with living ant colonies or abandoned attine nests. Two *Myrmecopterula* samples belonging to subclade SAPV1 (CALD170307–02 and CALD170307–03; Fig. 1a) were found forming basidiomes atop two distinct but adjacent (1 m apart) living *Apterostigma* nests in Amazonian Rainforest. The cultivated mutualists from both nests were also analysed and found to belong to *M. velohortorum* confirming that the basidiomes were not linked to the cultivated mycelia in these nests. The third member of subclade SAPV1 was also reported forming a nascent basidiome on a living *Apterostigma* nest in Panama (Munkacsi et al. 2004). *M. moniliformis* (SAPN1; Fig. 1e) has been reported to be found outside both active and apparently inactive (see *Myrmecopterula*: Notes on Taxonomy section above) attine nests (S. Sourell, pers. comm.; M.C. Aime, pers.comm.) as was CALD170315–04 (SAPV2; Fig. 1b) and CALD170122–04 (SAPV3; Fig. 1c). Lastly, the mycelium of one sample (JSP 07–03 B 5.1; SAPV3) was isolated from a living *Atta capiguara* nest by Pereira et al. (2016).

The observations above and the phylogenetic analyses suggests that association with attine ants is a widespread trait amongst members of this clade, hence its naming as *Myrmecopterula*.

Most recent attention on *Pterulaceae* has been lavished on the ant-cultivated mutualists *M. nudihortorum* and *M. velohortorum*. These were once thought to be sister clades (Munkacsi et al. 2004; Villesen et al. 2004) but are now known to be only distantly related within the *Myrmecopterula* clade (Dentinger et al. 2009, Fig. 3). This suggests two possibilities for the evolution of the *Myrmecopterula*-*Apterostigma* mutualism: (1) that it evolved independently on two occasions, or (2) that it is an ancestral condition of all *Myrmecopterula*. However, it is at present unclear whether the extant mutualistic association found for *M. nudihortorum* and *M. velohortorum* is ancestral, implying that the other taxa escaped the mutualism, or whether the looser association with ant nests widespread amongst members of *Myrmecopterula* was more recently elevated to a higher level of interdependence for these two species, as suggested by Dentinger et al. (2009). It is also possible that the free-living species within the *Myrmecopterula* may be specialised parasites specifically targeting their sister species that have formed a mutualism with the ants. An analogous situation is found in the leaf-cutting ants species *Acromyrmex echinatior* and its sister species *Acromyrmex insinuator*, the latter a highly specialised social parasite of the former (Sumner et al. 2004).

The basis of the association of ‘free-living’ species with attine ants and/or their abandoned nests is unclear. Given the apparent preference of some for abandoned nests, they may be specialised early stage colonisers of ant nest debris. A further possibility is that they are cheaters, deriving nutrition from the ant-collected biomass but not reciprocating by producing hyphae palatable to ants. This would represent a novel form of fungal mimicry, perhaps achieved by the ants’ inability to differentiate hyphae of closely related species. Lastly, they may be mycoparasitic, including on ant cultivars, although there is currently no direct evidence supporting this hypothesis.

### Re-delimitation of *Pterulaceae*

All the accepted genera in *Pterulaceae* were sampled in this study except for the monotypic *Allantula*. One specimen, with morphology consistent with Corner’s description of *Allantula diffusa*, with pteruloid basidiomes borne on slender mycelial cords as curved intercalary swellings, was collected during our fieldwork (Fig. 1m). Phylogenetic reconstruction placed this specimen firmly within *Phaeopterula*. However, we have been unable to obtain the type specimen (no other collections authenticated exist) for more detailed analysis.

Thus, we re-delimit *Pterulaceae* as containing six genera: *Allantula*, *Coronicium*, *Merulicium*, *Myrmecopterula*, *Phaeopterula*, *Pterula,* and *Pterulicium*.

## CONCLUSION

In this study, we presented a reclassification of *Pterulaceae* based on morphological and phylogenetic analyses with samples from six out of seven genera previously accepted in the family. Three early diverging resupinate genera were placed in the new family *Radulomycetaceae* (*Aphanobasidium*, *Radulomyces* and *Radulotubus*); the new genus *Myrmecopterula* was introduced to accommodate ant associated species previously classified in *Pterula*; several species from the latter were also recombined in the reintroduced *Phaeopterula* and in *Pterulicium*, and finally *Deflexula* was synonymised with *Pterulicium*. *Pterulaceae* was thus re-delimited to accommodate seven genera *Allantula*, *Coronicium*, *Merulicium*, *Myrmecopterula*, *Phaeopterula*, *Pterula* and *Pterulicium*. Some species kept in *Pterula* might also need to be recombined since the original description was not enough to make these changes. Type specimens should be analysed considering the delimitations proposed in this study.

## Supplementary information

**Additional file 1.** Full details of all samples studied here (simplified in Table 2; as excel file)

**Additional file 2.** Additional phylogenetic reconstructions, including detailed analyses relating to Fig. 3

**Additional file 3 **Additional images of coralloid *Pterulaceae* and micrographs of *Myrmecopterula velohortorum*.

## Abbreviations

BI: Bayesian inference
BPP: Bayesian posterior probability
CTAB: cetyltrimethylammonium bromide
DNA: deoxyribonucleic acid
EDTA: Ethylenediaminetetraacetic acid
ESS: Effective sample size
HPD: Highest posterior density
IUPAC: International Union of Pure and Applied Chemistry
ML: Maximum likelihood
nrITS: nuclear ribosomal internal transcribed spacer
nrLSU: nuclear ribosomal largre subunit
PCR: polymerase chain reaction
*Ph*.: *Phaeopterula*
*Pm*.: *Pterulicium*
PSRF: Potential scale reduction factors
*Pt*.: *Pterula*
RPB2: RNA polymerase B subunit 2
U: Protein unit
UFBoot: Ultrafast bootstrap

## Availability of data and materials

Details of the availability of the data and material used in this study can be found within the text.

DNA sequences were submitted to NCBI Genbank database (see Table 2 and Additional file 1). Alignments were deposited at TreeBase (ID: 24428). Dried specimens are deposited in the fungaria listed in the Methods section.
