# Supplementary material for: Reclassification of Pterulaceae Corner (Basidiomycota: Agaricales) introducing the ant-associated genus Myrmecopterula gen. nov., Phaeopterula Henn. and the corticioid Radulomycetaceae fam. nov
Source: IMA Fungus. 2020 Jan 30;11:2. doi: 10.1186/s43008-019-0022-6 (PMC7325140; doi:10.1186/s43008-019-0022-6)

**Main analyses for Figure 3**

**IQ-TREE Analysis**

1) IQ-TREE best scheme and substitution models searches, followed by best tree search and UFBoot with 1000 replicates.

1.1) Model-finder

Command:

DNA: iqtree -s $DNA_ALIGNMENT -spp partitions.txt -m MF+MERGE

INDEL: iqtree -s $INDEL_ALIGNMENT -st MORPH -m MF

Partition file:

begin sets;

charset ITS1_ITS2 = $DNA_ALIGNMENT: 1-265 427-658;

charset 58S_LSU = $DNA_ALIGNMENT: 266-426 657-1458;

charset RPB2 = $DNA_ALIGNMENT: 1459-2125;

charset indel = $INDEL_ALIGNMENT:MORPH , *;

charpartition mymodels =

TIM2+F+G4: ITS1_ITS2,

SYM+I+G4: 58S_LSU,

TIM3e+R3: RPB2,

MK+FQ+ASC+G4: indel;

end;

1.2) Analysis command

iqtree -spp $ALIGNMENT -bb 1000 -bspec GENESITE

2) Best scheme and substitution model search to implement on MrBayes

Command:

iqtree -s $ALIGNMENT -spp partitions.txt -m TESTMERGEONLY -mset mrbayes

Best_scheme result:

ITS1_ITS2: GTR+F+I+G4

58S_LSU: GTR+F+I+G4

RPB2: SYM+I+G4

INDEL: for indel partition we used a Standard morphological analyses with gamma distribution

**MrBayes Analysis**

For MrBayes analysis the following files were created. The parameters in the partitions file were retrieved from the IQ-TREE analysis 2 above.

**Supplementary analysis**

A dataset including all the sequences in Suppdata1 was created to find the best outgroup for Pterulaceae/Radulomycetaceae and to ensure that all the sequences from each cloned sample would cluster together.

The alignment and ML tree reconstruction followed the same methods for the main Figure 3, with exception of indel coding and trimming of ambiguous regions. And the best scheme result was:

ITS1_58S_ITS2_LSU_RPB2: TIM2+F+I+G4

The resulting tree (Suppdata2 Fig. 1) showed Sptephanosporaceae as the best outgroup for Pterulaceae/Radulomycetaceae and

**Suppdata2 Fig. 1 (below):** ML tree of Pterulaceae/Radulomycetaceae and several outgoups.


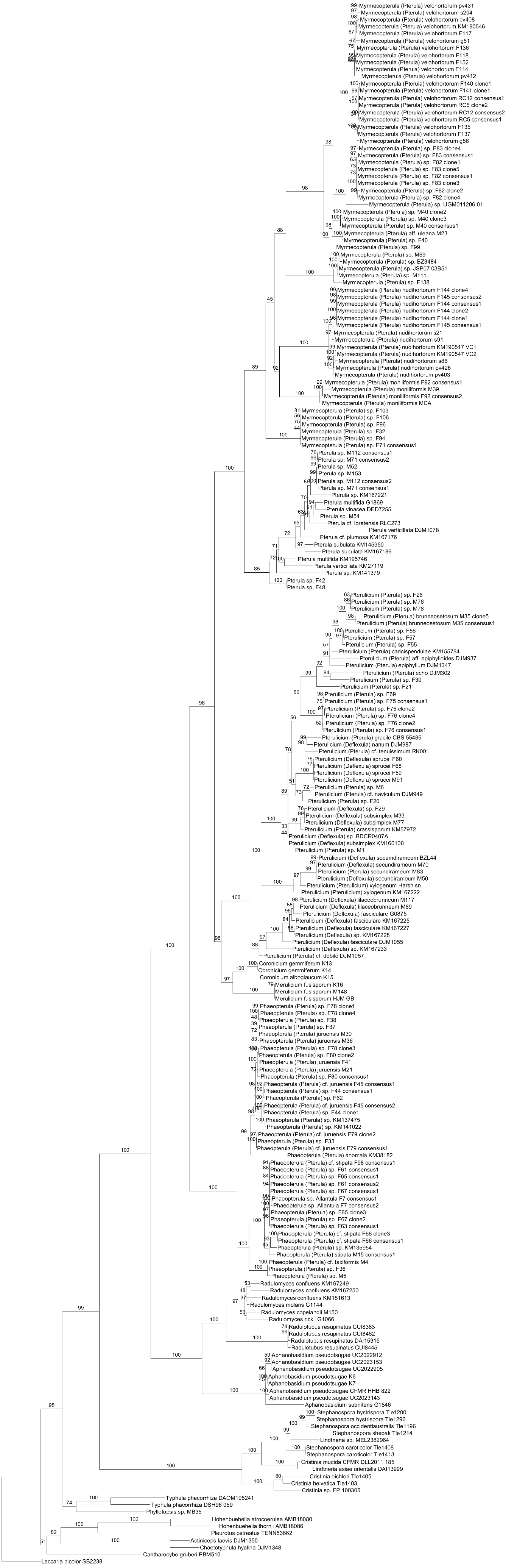


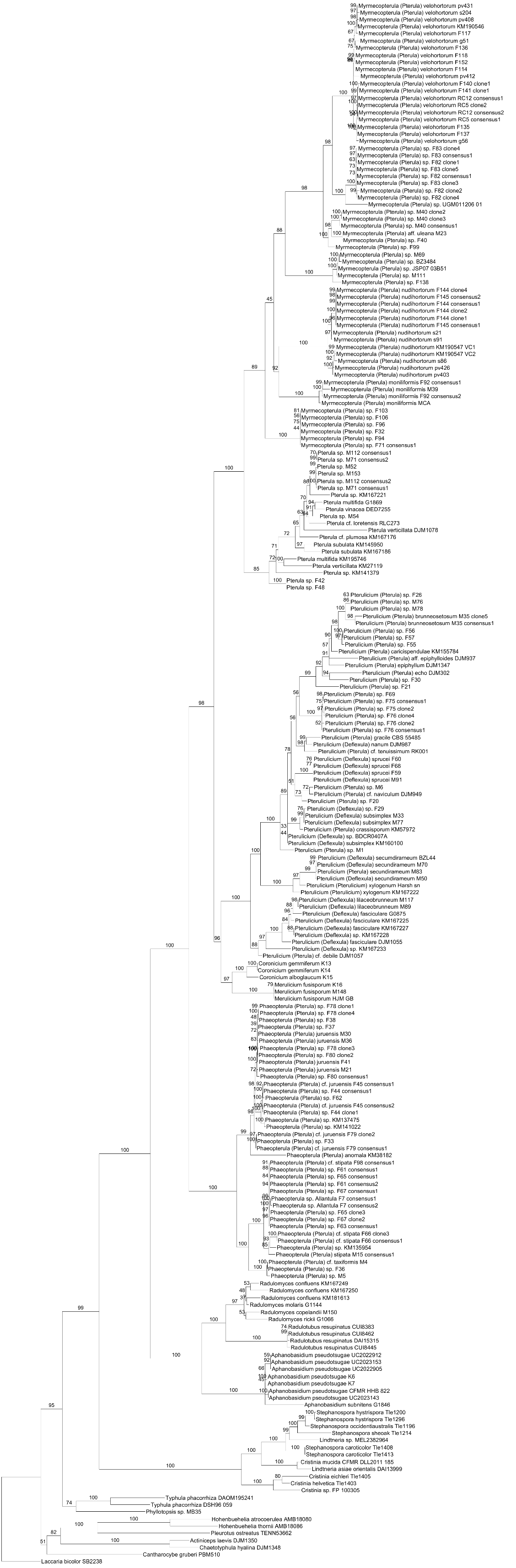

Supplement: Supplementary file 2 — Additional file 2. Additional phylogenetic reconstructions, including detailed analyses relating to Fig. 3 [file 43008_2019_22_MOESM2_ESM.docx]
