# Supplementary material for: Reclassification of Pterulaceae Corner (Basidiomycota: Agaricales) introducing the ant-associated genus Myrmecopterula gen. nov., Phaeopterula Henn. and the corticioid Radulomycetaceae fam. nov
Source: IMA Fungus. 2020 Jan 30;11:2. doi: 10.1186/s43008-019-0022-6 (PMC7325140; doi:10.1186/s43008-019-0022-6)
Supplement: Supplementary file 3 — Additional file 3 Additional images of coralloid Pterulaceae and micrographs of Myrmecopterula velohortorum. [file 43008_2019_22_MOESM3_ESM.pptx]

## Slide 1
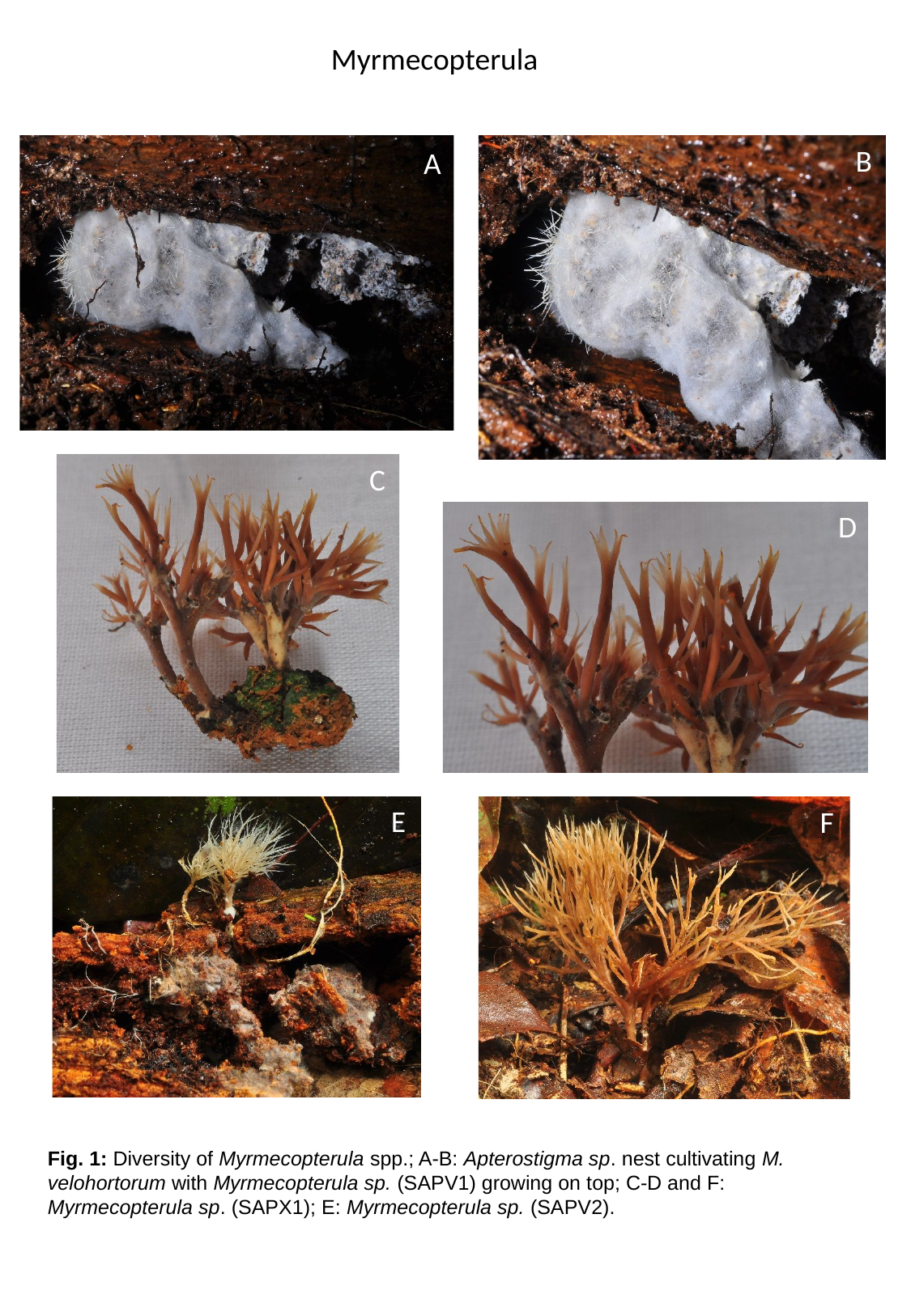

Myrmecopterula
B
A
C
D
E
F
Fig. 1: Diversity of Myrmecopterula spp.; A-B: Apterostigma sp. nest cultivating M. velohortorum with Myrmecopterula sp. (SAPV1) growing on top; C-D and F: Myrmecopterula sp. (SAPX1); E: Myrmecopterula sp. (SAPV2).

## Slide 2
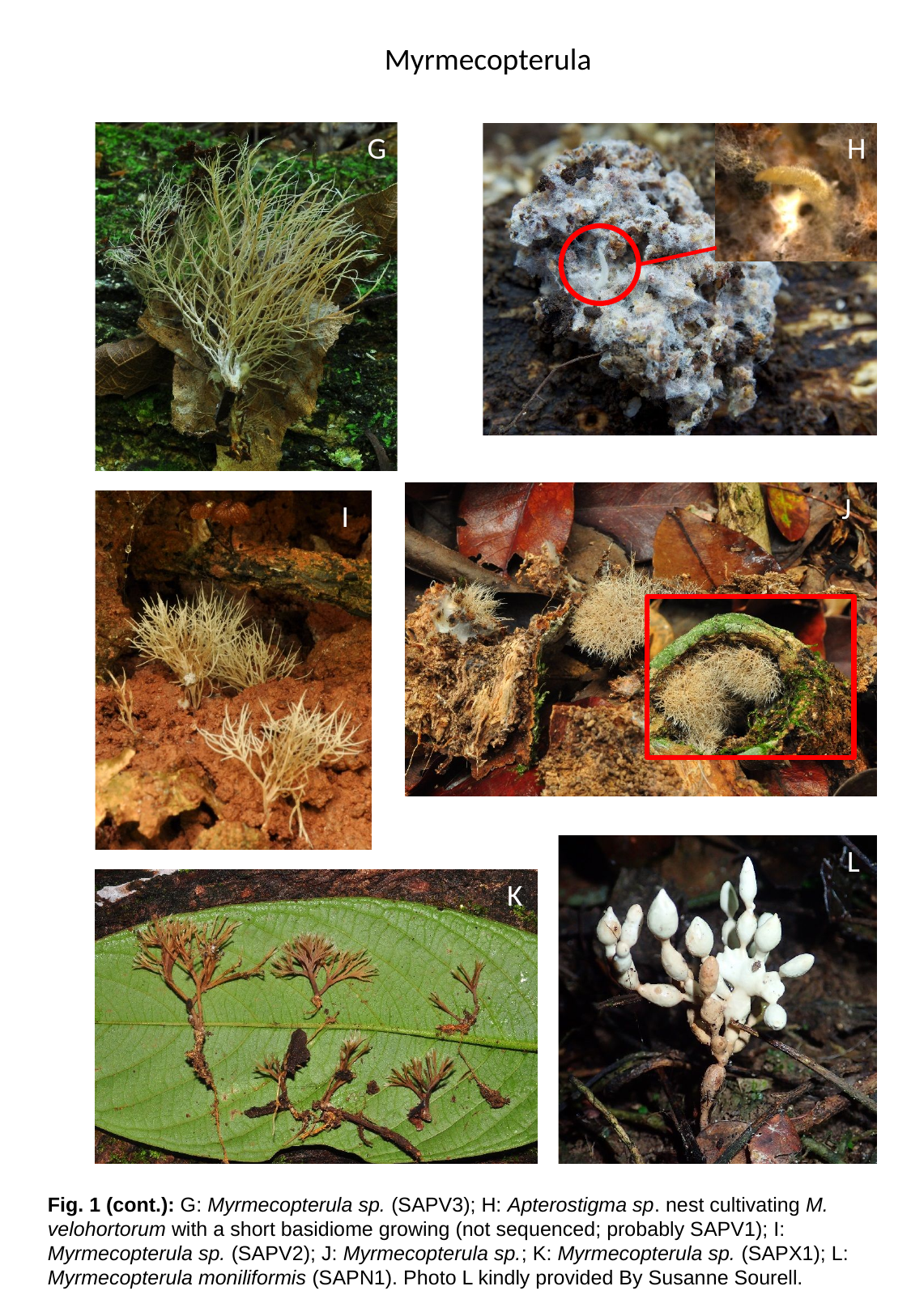

Myrmecopterula
G
H
J
I
L
K
Fig. 1 (cont.): G: Myrmecopterula sp. (SAPV3); H: Apterostigma sp. nest cultivating M. velohortorum with a short basidiome growing (not sequenced; probably SAPV1); I: Myrmecopterula sp. (SAPV2); J: Myrmecopterula sp.; K: Myrmecopterula sp. (SAPX1); L: Myrmecopterula moniliformis (SAPN1). Photo L kindly provided By Susanne Sourell.

## Slide 3
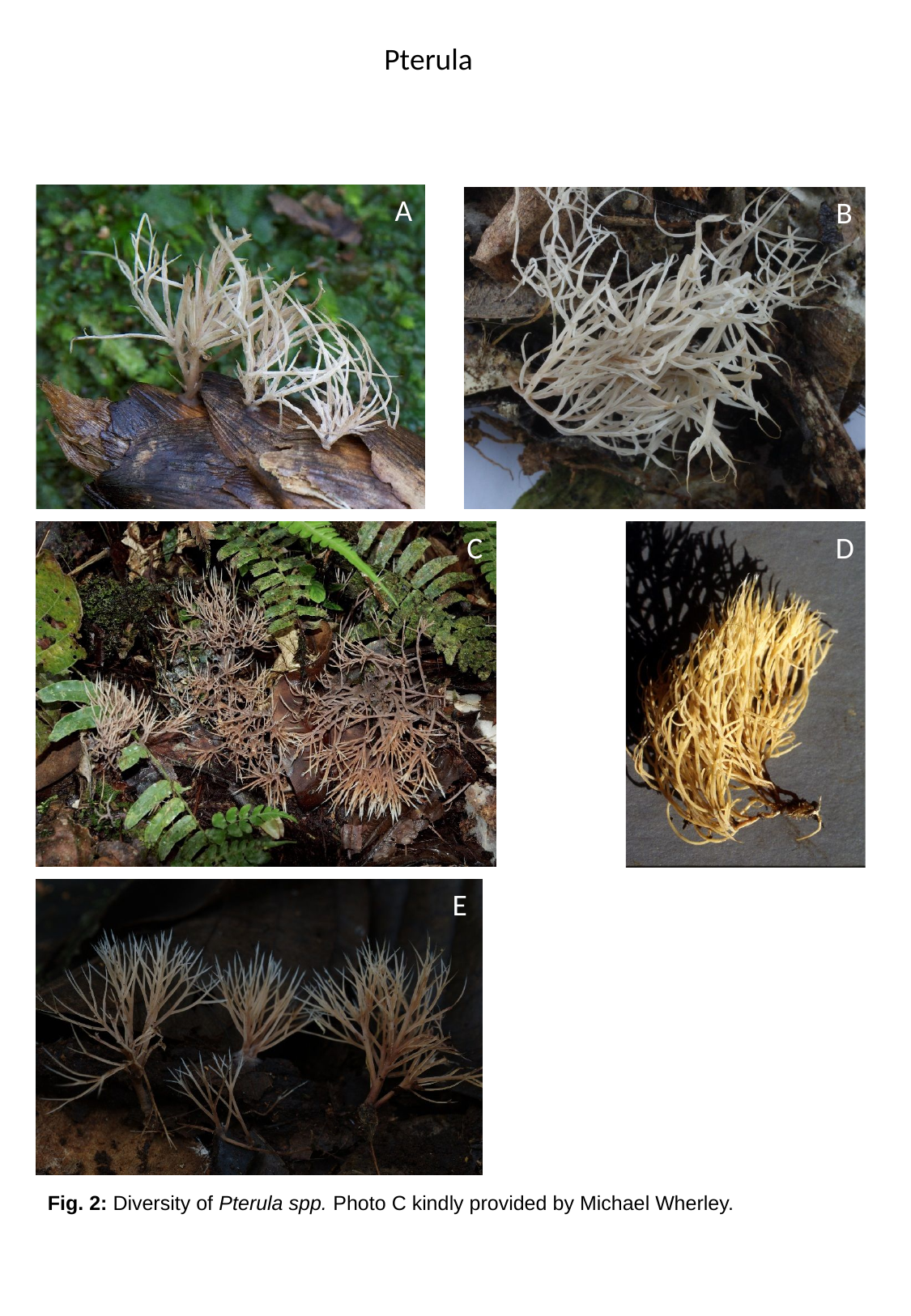

Pterula
A
B
C
D
E
Fig. 2: Diversity of Pterula spp. Photo C kindly provided by Michael Wherley.

## Slide 4
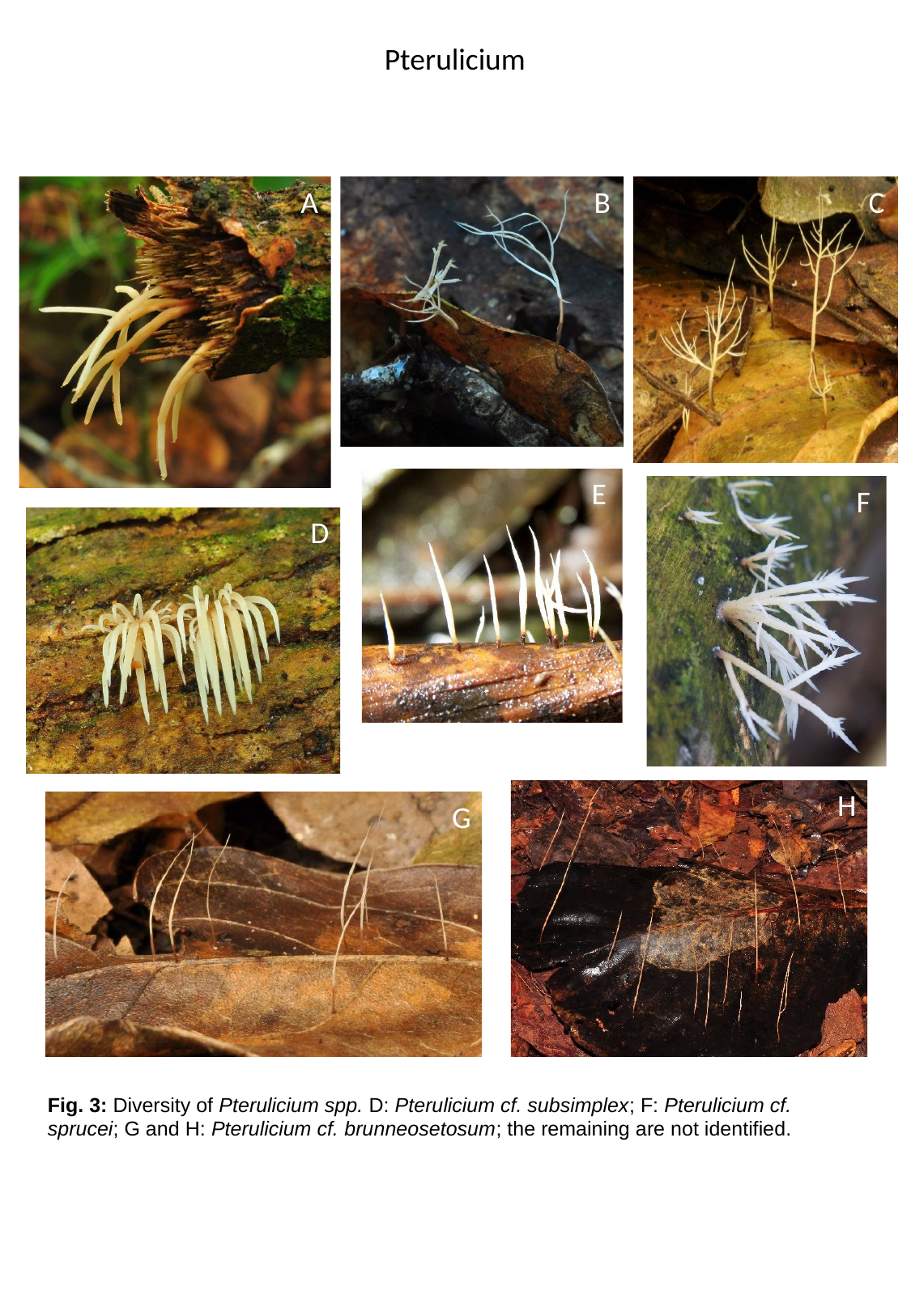

Pterulicium
A
B
C
E
F
D
H
G
Fig. 3: Diversity of Pterulicium spp. D: Pterulicium cf. subsimplex; F: Pterulicium cf. sprucei; G and H: Pterulicium cf. brunneosetosum; the remaining are not identified.

## Slide 5
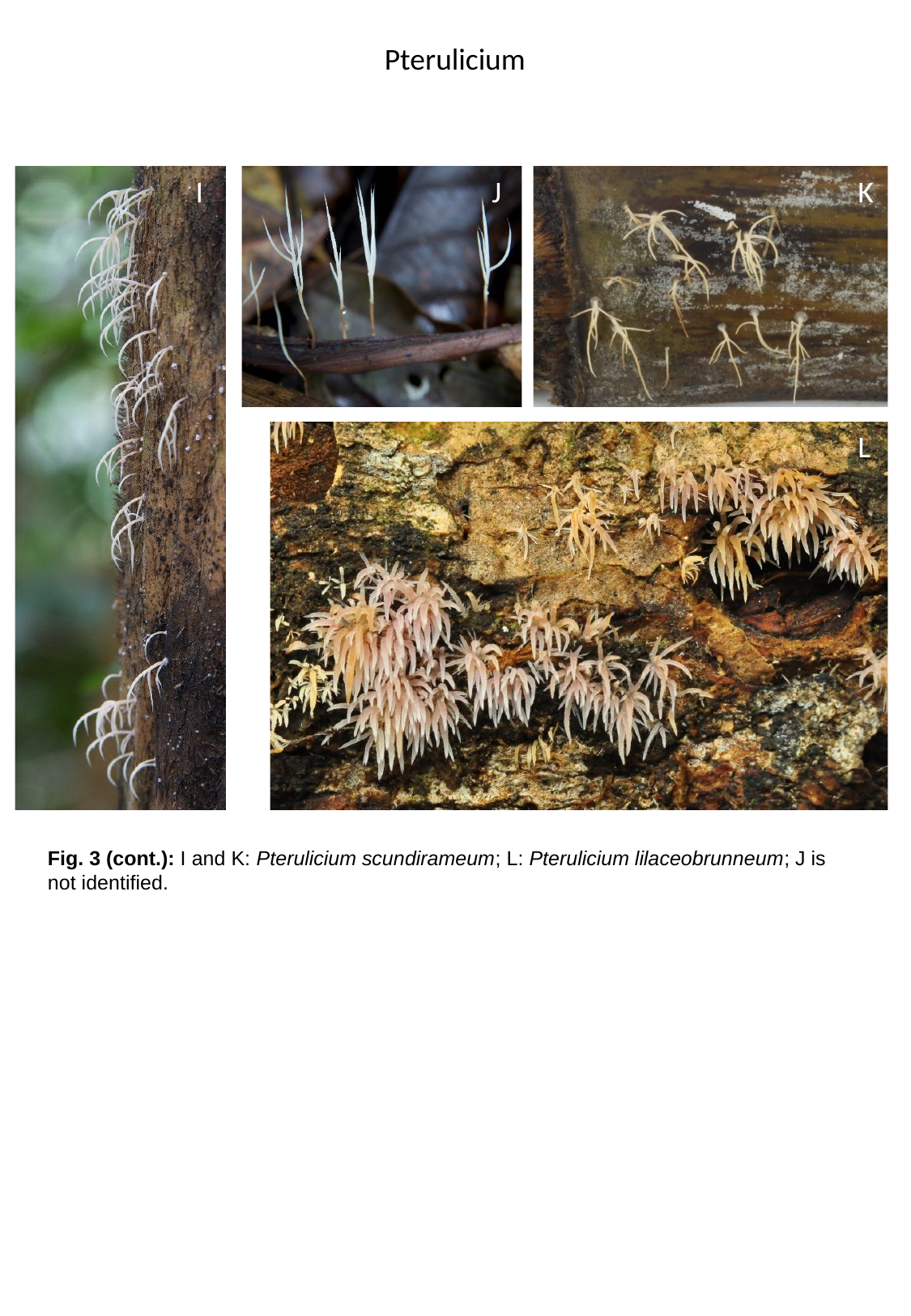

Pterulicium
I
J
K
L
Fig. 3 (cont.): I and K: Pterulicium scundirameum; L: Pterulicium lilaceobrunneum; J is not identified.

## Slide 6
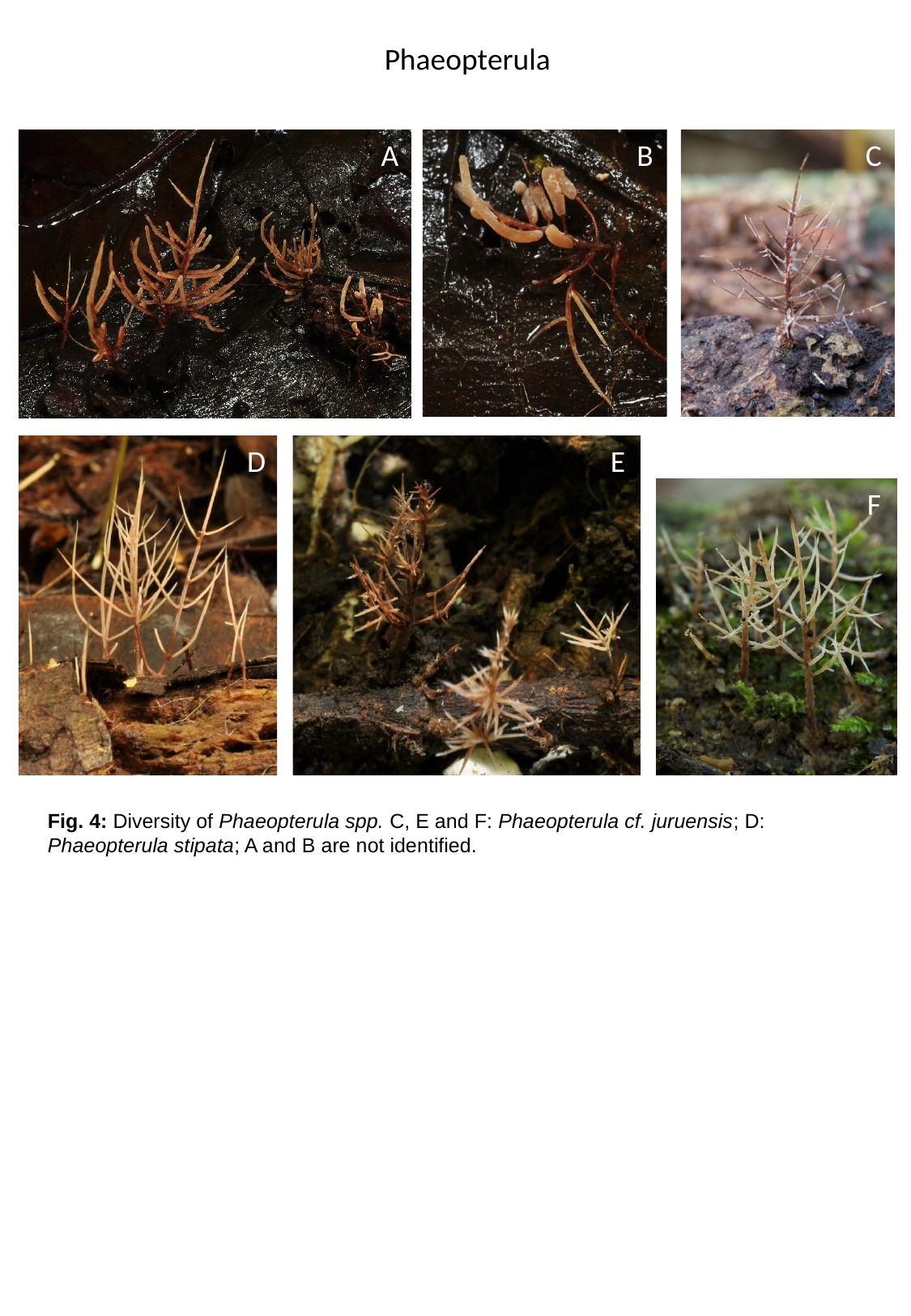

Phaeopterula
A
B
C
D
E
F
Fig. 4: Diversity of Phaeopterula spp. C, E and F: Phaeopterula cf. juruensis; D: Phaeopterula stipata; A and B are not identified.

## Slide 7
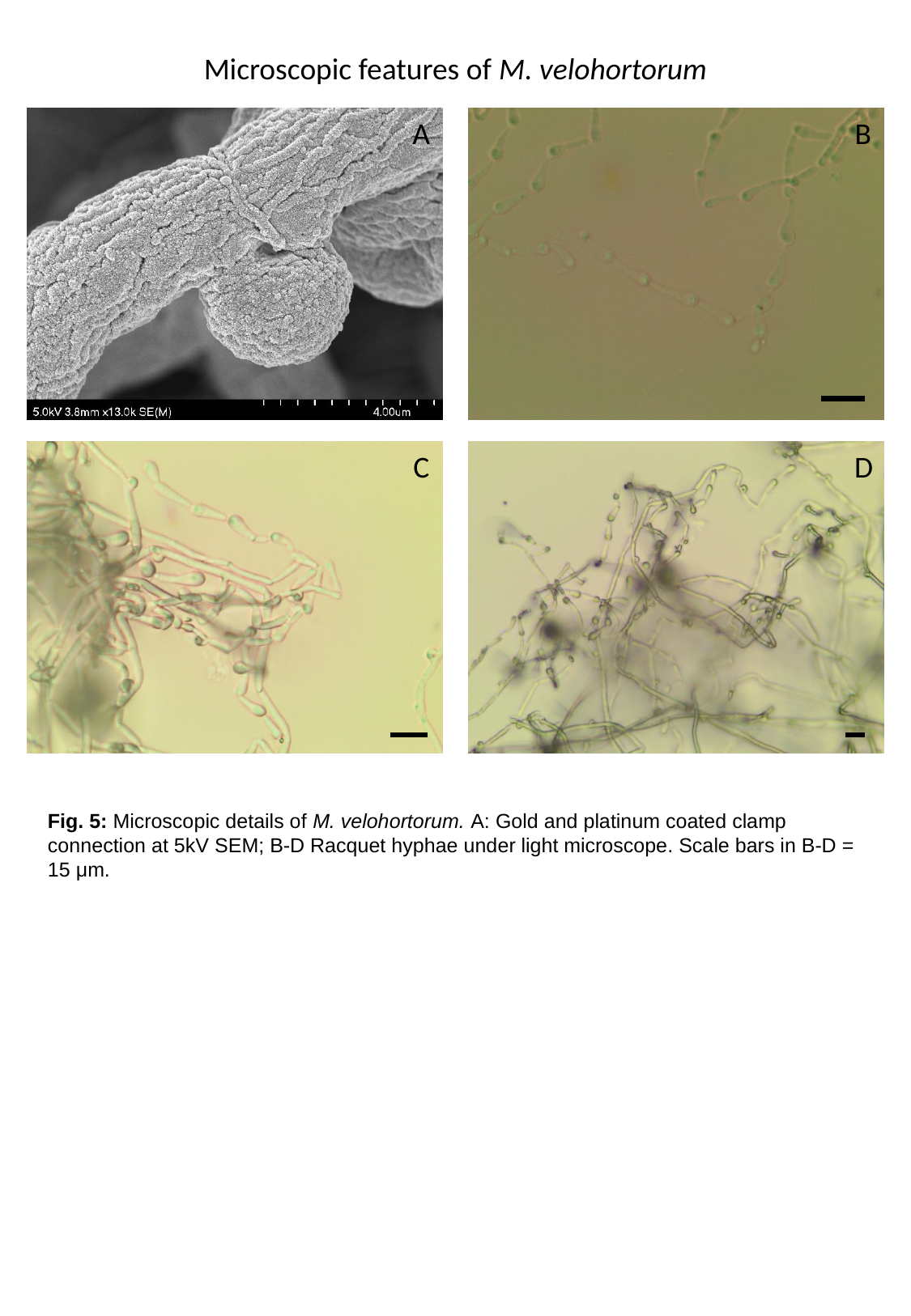

Microscopic features of M. velohortorum
A
B
C
D
Fig. 5: Microscopic details of M. velohortorum. A: Gold and platinum coated clamp connection at 5kV SEM; B-D Racquet hyphae under light microscope. Scale bars in B-D = 15 μm.
